# The incidence of fractures at various sites in newly treated patients with type 2 diabetes mellitus

**DOI:** 10.1016/j.bonr.2022.101614

**Published:** 2022-08-22

**Authors:** Cindy Sarodnik, Nicklas H. Rasmussen, Sandrine P.G. Bours, Nicolaas C. Schaper, Peter Vestergaard, Patrick C. Souverein, Morten H. Jensen, Johanna H.M. Driessen, Joop P.W. van den Bergh

**Affiliations:** a NUTRIM Research School, Maastricht University, Maastricht, the Netherlands; b Steno Diabetes Center North Jutland, Aalborg University Hospital, Aalborg, Denmark; c Department of Internal Medicine, Maastricht University Medical Centre+, the Netherlands; d CAPHRI Research School, Maastricht University, Maastricht, the Netherlands; e CARIM Research School, Maastricht University, Maastricht, the Netherlands; f Steno Diabetes Center North Jutland, Department of Endocrinology, Aalborg University Hospital, Aalborg, Denmark; g Division of Pharmacoepidemiology & Clinical Pharmacology, Utrecht Institute for Pharmaceutical Sciences, Utrecht University, Utrecht, the Netherlands; h Department of Clinical Pharmacy and Toxicology, Maastricht University Medical Centre+, Maastricht, the Netherlands; i Department of Internal Medicine, VieCuri Medical Center, Venlo, the Netherlands

**Keywords:** BMI, body mass index, *CPRD*, Clinical Practice Research Datalink, *IR*, incidence rate, *IRR*, incidence rate ratio, *ISAC*, Independent Scientific Advisory Committee, *MHRA*, Medicines and Healthcare products Regulatory Agency, *MOF*, major osteoporotic fracture, *NIAD*, non-insulin antidiabetic drug, *PY*, person year, *T2D*, type 2 diabetes mellitus, Type 2 diabetes, Newly treated type 2 diabetes, Incident fractures, Fracture pattern, Body mass index

## Abstract

**Purpose:**

In this descriptive study, we examined the incidence of fractures in patients with newly treated type 2 diabetes mellitus (T2D) compared to matched reference population.

**Methods:**

Participants from the UK Clinical Practice research datalink (CPRD) GOLD (1987–2017), aged ≥30 years, with a T2D diagnosis code and a first prescription for a non-insulin anti-diabetic drug (n = 124,328) were included. Cases with T2D were matched by year of birth, sex and practice to a reference population (n = 124,328), the mean follow-up was 7.7 years. Crude fracture incidence rates (IRs) and incidence rate ratios (IRRs) were calculated. Analyses were stratified by fracture site and sex and additionally adjusted for BMI, smoking status, alcohol use and history of any fracture at index date.

**Results:**

The IR of all fractures and major osteoporotic fractures was lower in T2D compared to the reference population (IRR 0.97; 95%CI 0.94–0.99). The IRs were lower for clavicle (IRR 0.67; 0.56–0.80), radius/ulna (IRR 0.81; 0.75–0.86) and vertebral fractures (0.83; 0.75–0.92) and higher for ankle (IRR 1.16; 95%CI 1.06–1.28), foot (1.11; 1.01–1.22), tibia/fibula (1.17; 1.03–1.32) and humerus fractures (1.11; 1.03–1.20). Differences in IRs at various fracture sites between T2D and the reference population were more pronounced in women than in men. In contrast, BMI adjusted IRs for all fractures (IRR 1.07; 1.04–1.10) and most individual fracture sites were significantly higher in T2D, especially in women.

**Conclusion:**

The crude incidence of all fractures was marginally lower in patients with newly treated T2D compared to the matched reference population but differed according to fracture site, especially in women. BMI adjusted analyses resulted in higher incidence rates in T2D at almost all fracture sites compared to crude incidence rates and this was more pronounced in women than in men. This implies that BMI may have a protective impact on the crude incidence of fractures, especially in women with newly treated T2D.

## Highlights

• Incidence of the total number of fractures was not higher in type 2 diabetic (T2D) patients during the first 8-9 years of disease.
• The distribution of these fractures did differ between patients and controls, in particular in the women.
• After correction for weight the incidence of the total number of fractures was increased in patients with T2D
• In T2D patients the elevated BMI can be a protective factor for developing (osteoporotic) fractures.

## 1 Introduction

Type 2 diabetes mellitus (T2D) is a common low-grade chronic inflammatory disease, characterized by an impaired glucose metabolism and the development of micro- and macrovascular complications. The worldwide prevalence of T2D was about 424.9 million in 2017 and it is expected to rise up to 628.6 million in 2045 (IDF, n.d.). Several studies suggested that T2D is associated with an increased fracture risk, although the aetiology of this increased risk is largely unknown (Bonds et al., 2006; Schwartz et al., 2001). The International Osteoporosis Foundation states that fracture risk might be increased due to a higher propensity of falls and decreased bone quality (Napoli et al., 2017).

Most epidemiological studies concerning fracture incidence in T2D mainly focussed on the overall fracture risk, and often a 1.2–2.4 fold increased risk is reported (Bonds et al., 2006; Holmberg et al., 2006). Studies focusing on multiple fracture sites in T2D are limited and results are inconsistent due to different study designs, study populations, gender distributions, definition of diabetes, diabetes duration and severity (Bonds et al., 2006; Schwartz et al., 2001; Zhang et al., 2019; Wang et al., 2019; Moayeri et al., 2017; Vestergaard, 2007). An important limitation of these studies is that a case-mix of patients with prevalent and incident T2D were included, as it is conceivable that only during the course of the disease the risk of fractures slowly increases, in parallel to and possibly as a result of other diabetic complications and as a result of the effects of long term hyperglycaemia on bone. Therefore, it is unclear whether the risk of future fractures is increased due to the diabetic state per se or that this risk is more related to the development of long-term complications and/or long-term hyperglycaemia. The question that remains to be answered is therefore if the risk of fracture is already increased at an early stage of T2D and whether this differs among various fracture sites? Additionally, it remains uncertain if the fracture pattern in T2D differs by sex, since several earlier studies focussed on fracture incidence in women rather than in men (Bonds et al., 2006; Schwartz et al., 2001; Dytfeld and Michalak, 2017). Studies that stratified participants by sex often showed that women with T2D had a higher fracture incidence compared to the reference population whereas this was not always found in men with T2D (Holmberg et al., 2006; Ahmed et al., 2006). Finally, since most patients with T2D are obese, obesity may also affect the risk of fractures and its pattern in patients with T2D; obesity may be protective for some fracture types and a risk factor for other fracture types.

We hypothesized that fracture incidence will differ among the various fracture locations in patients with T2D compared to the matched reference population, especially in women and that obesity may be protective for fractures at specific sites and therefore might attenuate fracture risk in T2D.

In this descriptive study, we aimed to study the pattern of incident fractures according to fracture site in patients with newly treated T2D compared to the matched reference population. Additionally, we aimed to determine whether the fracture pattern was different for men and women and whether the association of T2D with fracture risk is attenuated by the presence of obesity.

## 2 Methods

### 2.1 Data source

We conducted a descriptive population-based retrospective cohort study using data of the UK Clinical Practice Research Datalink (CPRD) GOLD. The CPRD contains anonymized electronic medical records of 674 primary care practices in the United Kingdom, representing approximately 6.9 % of the total UK population in 2013 (Herrett et al., 2015). The data recorded in CPRD include patient demographics, medical history, laboratory test results, prescription details, specialist referrals, hospital admissions and major outcomes since 1987, with on-going data collection (Herrett et al., 2015). The population within the CPRD is widely representative for the UK population and it was reported that the accuracy and completeness of data, especially about age and sex, is satisfactory (Herrett et al., 2010; Khan et al., 2010). Our study protocol (Protocol 18_275R) was approved as a descriptive study by the Independent Scientific Advisory Committee (ISAC) for Medicines and Healthcare products Regulatory Agency (MHRA) database research. Informed consent was not required in this study, since all data on patients are stored anonymously in CPRD.

### 2.2 Study population

**Fig. 1:**
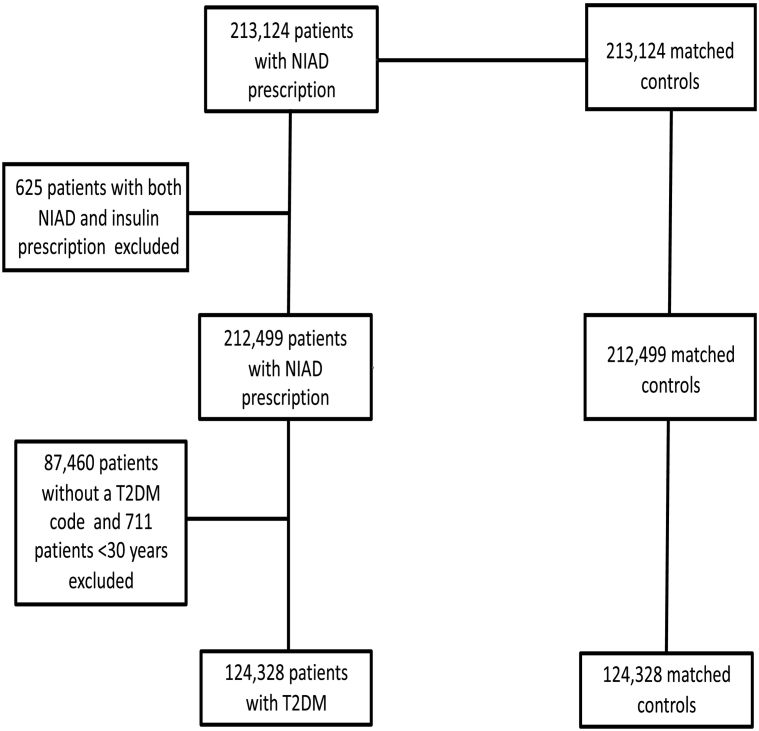
Flow chart of selection of the study population; when a patient with T2D was excluded, his matched reference individual was excluded as well.

Our T2D cohort consisted of all patients with T2D who received a first ever prescription of a non-insulin anti-diabetic drug (NIAD), after their date of T2D diagnosis, between January 1, 1987 and December 31, 2017. The date of the first ever NIAD prescription determined the index date. Each case with T2D was matched by year of birth, sex and practice to a patient without a T2D diagnosis and without a prescription of a NIAD or insulin (a reference patient) using incidence density sampling (Richardson, 2004). In order to identify patients with incident T2D, patients needed to have at least one year of valid data collection before the index date. The reference population were assigned the index date of their matched cases with T2D. The reference population were censored when they received a NIAD or insulin prescription or when a diabetes diagnosis Read code was recorded. A Read code is a clinical code that is used in primary care in the UK to registrate several medical events such as a diagnosis of a disease. Patients with T2D receiving insulin on or before the index date were excluded as well as patients receiving NIAD without a T2D diagnosis or patients aged <30 years. The selection of our final study population is shown in Fig. 1.

### 2.3 Demographics

The following demographics were determined at the index date: age, sex, body mass index (BMI), smoking status alcohol use. We determined history of fracture(s), history of major osteoporotic fracture (MOF) and history of diagnosis of osteoporosis before index date and use of anti-osteoporotic drugs 6 months prior to index date based on Read codes (diagnosis) and product codes (medication use).

### 2.4 Outcome

T2D cases and the reference population were followed from their index date up to the date of death, the end of data collection, the end of the study period, or the date of fracture (for every fracture site), whatever came first. Fractures were identified using Read codes and broken down by the following sites: ankle, wrist, clavicle, femoral region, foot, hip, humerus, patella, pelvis, radius/ulna, ribs, scapula, skull, tibia/fibula and vertebrae. Additional outcomes included all fractures, which is the first incident fracture at any site after index date. Femoral fractures were defined as all types of femoral fractures, open and closed fractures, except for hip fractures. Vertebral fractures were defined as clinical vertebral fractures within this study. A high level of validity of hip and vertebral fractures within the CPRD database has been reported previously (Van Staa et al., 2000). For example, the positive predictive value for vertebral fractures in CPRD was reported to be 88.1 % (81.3–93.0 %) (Khan et al., 2010).

### 2.5 Statistical analysis

Baseline characteristics are presented as mean (SD) for continuous variables and as number of patients (%) for categorical variables. Standardized differences (STD, difference in means of a variable divided by the pooled standard deviation) were calculated for baseline characteristics that were not matched for; a standard difference less than 0.1 was used as cut-off point to indicate a negligible difference between patients and matched the reference population (Austin, 2011). Fracture site and sex-specific crude incidence rates (IRs) were calculated by dividing the number of fractures by the total number of person years. Additionally, a Poisson model with Wald confidence limits was used to calculate the crude incidence rate ratios (IRRs) and 95 % confidence intervals (95%CI) between cases with T2D and the reference population (Rothman et al., 2008). Additionally the IRs (overall and the gender specific) were adjusted for BMI, smoking status, alcohol use, history of any fracture at index date and history of diagnosis of osteoporosis at index date or a prescription for anti-osteoporosis medication in the 6 months prior to the index date using a Cox proportional Hazards regression model. The likelihood ratio test was performed to check which model fits better. Furthermore we tested whether these adjustments resulted in statistically significantly different IRRs compared to the crude IRRs (Hoffmann et al., 2008). To take the matched design into account, a robust sandwich covariance matrix was used, when estimating the adjusted IRRs. As a sensitivity analysis we excluded all participants with a prescription for anti-osteoporosis medication in the six months prior to the index date or with diagnosis of osteoporosis prior to the index date from the main analysis. All statistical analyses were performed in SAS 9.4 (SAS Institute Inc. Cary, NC, USA).

## 3 Results

The baseline characteristics of our study population are shown in Table 1. We included 124,328 patients with T2D and 124,328 the matched reference population. The median time-gap between diabetes diagnosis and the date of the first NIAD was less than a year; 341 days with an interquartile range of 41–1127 days.

**Table 1:** Baseline characteristics of patients with T2D and the matched reference population.

	T2D (n = 124,328)		Reference population (n = 124,328)		Standardized differences
	Absolute numbers	%	Absolute numbers	%	
Mean follow-up time (years, SD)	7.7	4.9	7.4	4.9	0.05
Women	54,411	43.8	54,411	43.8	
Age (years, mean ± SD)	62.9 ± 12.5		62.9 ± 12.5		
Age groups					
30–39 years	4,007	3.2 %	4,007	3.2 %	
40–49 years	15,168	12.2 %	15,168	12.2 %	
50–59 years	29,692	23.9 %	29,692	23.9 %	
60–69 years	36,056	29.0 %	36,056	29.0 %	
70–79 years	27,822	22.4 %	27,822	22.4 %	
80+ years	11,583	9.3 %	11,583	9.3 %	
BMI					
Mean BMI (kg/m^2^ ± SD)	31.7 ± 6.5		27.0 ± 5.0	5.0	0.81
<20 kg/m^2^	1,161	0.9 %	5315	4.3 %	0.92
20–24.9 kg/m^2^	13,657	11.0 %	35,620	28.7 %	
25–29.9 kg/m^2^	39,943	32.1 %	44,878	36.1 %	
30–34.9 kg/m^2^	36,602	29.4 %	18,224	14.7 %	
≥35 kg/m^2^	31,603	25.4 %	7260	5.8 %	
Obesity^a^	68,205	54.8 %	25,484	20.5 %	0.76
Missing	1362	1.1 %	13,031	10.5 %	
Smoking status					0.32
Never	36,596	29.4 %	44,927	36.1 %	
Former	62,834	50.5 %	48,581	39.1 %	
Current	24,576	19.8 %	26,292	21.1 %	
Missing	322	0.3 %	4528	3.6 %	
Alcohol use					0.36
No	33,271	26.8 %	22,218	17.9 %	
Yes	87,644	70.5 %	89,192	71.7 %	
Missing	3413	2.7 %	12,918	10.4 %	
History of fracture(s)	26,064	21.0 %	26,488	21.3 %	−0.01
History of MOF	9170	7.4 %	10,077	8.1 %	−0.03
Osteoporosis					
Use of anti-osteoporotic drugs in 6 months prior to index date	6829	5.5 %	6511	5.2 %	−0.01
History of diagnosis of osteoporosis	2729	2.2 %	4237	3.4 %	−0.07
Use of anti-osteoporotic drugs in 6 months prior to index date or diagnosis of osteoporosis	7532	6.1 %	8116	6.5 %	−0.02

Categorical co-variates are presented as number of participants (%) and continues variables are presented as mean (SD). *BMI* body mass index, *MOF* major osteoporotic fracture, *SD* standard deviation, *T2D* type 2 diabetes mellitus.

a Obesity is defined as a BMI ≥ 30 kg/m^2^.

The proportion of women was 43.8 % and mean age was 62.9 ± 12.5 years in both cohorts. Patients with T2D had a higher mean BMI (31.7 kg/m^2^ versus 27.0 kg/m^2^, STD 0.81) and a higher proportion of former smokers (50.5 % versus 39.1 %, STD 0.32). History of fracture(s) (21.0 % versus 21.3 %, STD −0.01) or MOF (7.4 % versus 8.1 %, STD −0.03) was similar at baseline between patients with T2D and the reference population. The use of anti-osteoporotic drugs in 6 months prior to index date was higher in patients with T2D (5.5 % versus 5.2 %), whereas the number of participants with a history of diagnosis of osteoporosis was higher in the reference population (3.4 % versus 2.2 %). The mean duration of follow-up was 7.7 ± 4.9 years (in total 952,505 person years) in the T2D cohort and 7.4 ± 4.9 years (in total 920,012 person years) in the reference cohort with a standardized difference (STD) of 0.05.

**Fig. 2:**
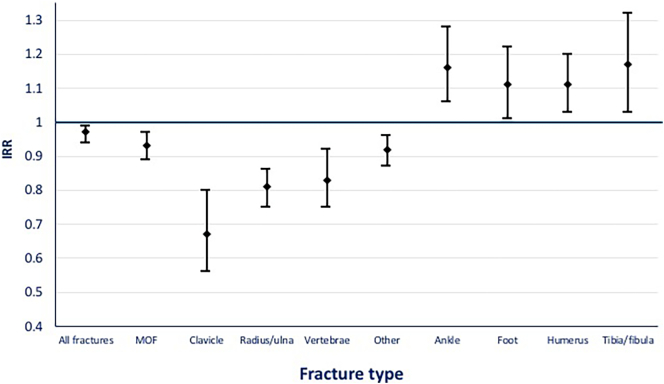
Fracture sites with different incidence rate ratios in patients with T2D compared to their matched- reference population. IRR incidence rate ratio, CI confidence interval.

Table 2 shows the IRs and IRRs of fractures in patients with T2D and the reference population. Patients with T2D had a lower incidence for all fractures compared to the reference population (IR: 10.9 and 11.3 per 1000 PYs, respectively, IRR 0.97; 95%CI 0.94–0.99). When analysing the different fracture sites separately, a significantly lower IR was observed in the T2D cohort for fractures at the clavicle (IRR 0.67; 0.56–0.80), radius/ulna (IRR 0.81; 0.75–0.86) and vertebrae (IRR 0.83; 0.75–0.92) (Fig. 2). In contrast, the IRs was higher in the T2D cohort for fractures at the ankle (IRR 1.16; 1.06–1.28), foot (IRR 1.11; 1.01–1.22), humerus (IRR 1.11; 1.03–1.20) and tibia/fibula (IRR 1.17; 1.03–1.32) (Fig. 2). The incidence rates of fractures at the other fracture sites were not significantly different between the T2D and the reference cohort.

**Table 2:** The incidence rates and incidence rate ratios for different fracture sites in patients with T2D versus the matched reference population.

*Fracture site*	T2D (n = 124,328)		Reference population (n = 124,328)		IRR T2D/reference population (CI 95 %)
	Number of fractures	IR (/1000 PYs)	Number of fractures	IR (/1000 PYs)	
Ankle	917	1.0	763	0.8	1.16 (1.06–1.28)^a^
Foot	891	0.9	778	0.9	1.11 (1.01–1.22)^a^
Humerus	1412	1.5	1226	1.3	1.11 (1.03–1.20)^a^
Tibia/fibula	524	0.6	434	0.5	1.17 (1.03–1.32)^a^
All	9832	10.9	9793	11.3	0.97 (0.94–0.99)^a^
Clavicle	202	0.2	290	0.3	0.67 (0.56–0.80)^a^
Radius/Ulna	1533	1.6	1834	2.0	0.81 (0.75–0.86)^a^
Vertebrae	679	0.7	789	0.9	0.83 (0.75–0.92)^a^
Femur	221	0.2	240	0.3	0.89 (0.74–1.07)
Carpal	1131	1.2	1098	1.2	0.99 (0.92–1.08)
Hip	1557	1.6	1566	1.7	0.96 (0.89–1.03)
Patella	108	0.1	108	0.1	0.97 (0.74–1.26)
Pelvis	368	0.4	396	0.4	0.90 (0.78–1.03)
Ribs	555	0.6	498	0.5	1.08 (0.95–1.21)
Scapula	114	0.1	109	0.1	1.01 (0.78–1.31)
Skull	246	0.3	239	0.3	0.99 (0.83–1.19)

*CI* confidence interval, *IR* incidence rate, *IRR* incidence rate ratio, *PYs* person years, *T2D* type 2 diabetes mellitus.

a Statistically significant results.

In the adjusted analyses, IRRs changed substantially for several fracture sites (Supplementary Table 1) with a reversal of the seemingly ‘protective’ effect of T2D on fracture incidence. After adjustment for BMI the IR for all fractures (IRR 1.07; 1.04–1.10), hip (IRR 1.44; 1.33–1.55), humerus (IRR 1.20; 1.10–1.30), pelvis (IRR 1.23; 1.05–1.44), ribs (IRR 1.25; 1.09–1.42) and tibia/fibula (IRR 1.19; 1.03–1.37) fractures was significantly higher in the T2D cohort compared to the reference cohort, while the IR of radius/ulna fractures (IRR 0.88; 0.82–0.95) was significantly lower. After additional adjustment for smoking status, alcohol use, history of any fracture at index date and history of diagnosis of osteoporosis at index date or a prescription for anti-osteoporosis drugs in the six months prior to the index date, IRs and IRRs were quite comparable with the analyses adjusted only for BMI. The likelihood ratio test was performed, to see which model is fitting better, and for most outcomes it gave a significant better fit when adding the adjustments instead of the crude model (data not shown).

When comparing BMI adjusted IRRs with the crude IRRs, BMI adjusted IRRs were significantly higher than crude IRRs (Supplementary Table 1) for all fractures (+10.3 %) and most other fracture sites, except that the BMI adjusted IRR's were lower for ankle (−12.1 %) and foot fractures (−6.3 %) and unchanged for tibia/fibula, carpal and scapula fractures. After additional adjustment for smoking status, alcohol use and history of any fracture at index date, results were comparable.

Table 3 shows the crude IRs and IRRs of fractures for men or women separately, with and without T2D. Compared to female reference population, women with T2D had statistically significantly lower IRs for all fractures (IRR 0.95; 95%CI 0.91–0.98), with lower IR's for several fracture sites as shown in the table. In men with T2D no such difference was observed in all fractures (IRR 1.01; 0.96–1.05) and lower IRs were not observed in any specific fracture site, except for hip fractures (IRR 0.88; 0.78–1.00). The IR of ankle fractures was higher both for women with T2D (IRR 1.15; 1.02–1.30) and men (IRR 1.20; 1.03–1.40), whereas the IR of humerus fractures was only higher in women with T2D (IRR 1.11; 1.01–1.21) and the IR of foot fractures was only higher in men with T2D (IRR 1.18; 1.01–1.38).

**Table 3:** The incidence rates and incidence rate ratios for different fracture sites in women and men with T2D versus women and men without T2D.

*Fracture site*	Women of T2D cohort (n = 54,411)		Women of reference cohort (n = 54,411)		IRR T2D/reference population (CI 95 %)	*Fracture site*	Men of T2D cohort (n = 69,917)		Men of reference cohort (n = 69,917)		IRR T2D/reference population (CI 95 %)
	Number of fractures	IR (/1000 PYs)	Number of fractures	IR (/1000 PYs)			Number of fractures	IR (/1000 PYs)	Number of fractures	IR (/1000 PYs)	
Ankle	562	1.4	479	1.2	1.15 (1.02–1.30)^a^	Ankle	355	0.7	284	0.6	1.20 (1.03–1.40)^a^
Humerus	974	2.4	860	2.1	1.11 (1.01–1.21)^a^	Humerus	438	0.8	366	0.7	1.15 (1.00–1.32)
All	5972	15.4	6123	16.2	0.95 (0.91–0.98)^a^	All	3860	7.5	3670	7.5	1.01 (0.96–1.05)
Clavicle	83	0.2	123	0.3	0.66 (0.50–0.87)^a^	Clavicle	119	0.2	167	0.3	0.68 (0.54–0.86)^a^
Radius/ulna	1123	2.7	1411	3.5	0.77 (0.71–0.84)^a^	Radius/ulna	410	0.8	423	0.8	0.93 (0.81–1.06)
Vertebrae	403	1.0	480	1.2	0.82 (0.72–0.93)^a^	Vertebrae	276	0.5	309	0.6	0.86 (0.73–1.01)
Femur	146	0.3	165	0.4	0.86 (0.69–1.08)	Femur	75	0.1	75	0.1	0.96 (0.70–1.32)
Carpal	615	1.5	597	1.5	1.01 (0.90–1.12)	Carpal	516	1.0	501	1.0	0.99 (0.87–1.12)
Foot	544	1.3	496	1.2	1.07 (0.95–1.21)	Foot	347	0.7	282	0.6	1.18 (1.01–1.38)^a^
Hip	1071	2.6	1040	2.6	1.00 (0.92–1.09)	Hip	486	0.9	526	1.0	0.88 (0.78–1.00)^a^
Patella	71	0.2	63	0.2	1.10 (0.78–1.54)	Patella	37	0.1	45	0.1	0.79 (0.51–1.22)
Pelvis	270	0.6	283	0.7	0.93 (0.79–1.10)	Pelvis	98	0.2	113	0.2	0.83 (0.63–1.09)
Ribs	213	0.5	211	0.5	0.99 (0.81–1.19)	Ribs	342	0.6	287	0.6	1.14 (0.98–1.34)
Scapula	60	0.1	58	0.1	1.01 (0.70–1.45)	Scapula	54	0.1	51	0.1	1.01 (0.69–1.49)
Skull	106	0.3	113	0.3	0.92 (0.70–1.19)	Skull	140	0.3	126	0.2	1.06 (0.84–1.35)
Tibia/fibula	299	0.7	256	0.6	1.14 (0.96–1.35)	Tibia/fibula	225	0.4	178	0.3	1.21 (1.00–1.47)

*CI* confidence interval, *IR* incidence rate, *IRR* incidence rate ratio, *PYs* person years, T2D type 2 diabetes mellitus.

a Significant results between women or men with T2D as compared to women or men without T2D.

When determining the effect of sex in the adjusted analyses, results in women (Supplementary Table 2A) were quite comparable to the results of the total population. The BMI adjusted IRRs of all fractures and of almost all other fractures sites were in women significantly higher compared to the crude IRR. In men no difference was observed in the BMI adjusted IRs of all fractures (Supplementary Table 2B), but those of the hip (IRR 1.18; 1.03–1.35), humerus (IRR 1.21; 1.04–1.40) and rib fractures (IRR 1.23; 1.04–1.46) were significantly higher in the men with T2D cohort compared to the male reference population, while the IRs for the other fracture sites were not significantly different. After additional adjustment for smoking status, alcohol use, history of any fracture, history of diagnosis of osteoporosis at index date and a prescription for anti-osteoporosis drugs in the six months prior to the index date results were comparable, except for the adjusted IR of hip fractures that was no longer different between patient with T2D and the reference population. In addition, the BMI adjusted (and the additional adjusted) IRR of all, clavicle, femur, hip, pelvis, ribs and vertebrae were significantly higher and IRR of ankle and foot fractures, were significantly lower in men compared to the crude IRR. The likelihood ratio test was also performed in the analyses for men and women separately, to see which model is fitting better, and for most outcomes it gave a significant better fit when adding the adjustments instead of the crude model (data not shown).

A sensitivity analysis showed that, after exclusion of participants with anti-osteoporosis treatment or a diagnosis of osteoporosis, the IRs and IRRs of fractures in patients with T2D and reference population were comparable to the results of the whole cohort, including participants with anti-osteoporosis treatment and a diagnosis of osteoporosis (data not shown). Only the IRs of vertebrae, and all fractures were not significantly different instead of marginally lower, after exclusion of participants with anti-osteoporosis treatment or a diagnosis of osteoporosis in participants with T2DM compared to the reference population.

## 4 Discussion

In this study, we examined crude and adjusted incidence rates according to fracture site in patients with newly treated T2D compared to the matched reference population For the total cohort, the crude incidence rate of all fractures was found to be marginally lower in the T2D group, although we consider this not a clinically relevant effect size. However, incidence rates varied considerably according to fracture site with a lower crude IR in the T2D cohort for fractures at the clavicle, radius/ulna and vertebral fractures while the crude IR was higher for fractures at the ankle, foot, humerus and tibia/fibula. These findings were mainly determined by the fracture pattern of women with T2D while the IRs in men with and without T2D were quite comparable. Importantly, the BMI adjusted IRs of all fractures combined and several specific fracture sites were significantly higher in patients with T2D compared to the reference population and only the adjusted IR of radius/ulna fractures was lower in T2D. In addition, almost all BMI adjusted IRRs were significantly higher than crude IRRs and further adjustments did not significantly change the results. These findings imply that BMI has a significant impact when analysing crude and adjusted facture incidence rates in patients with newly treated T2D.

Most previously published studies on fracture risk in T2D reported adjusted results without providing crude incidence or absolute risk data (Bonds et al., 2006; Ivers et al., 2001; Oei et al., 2013). Although adjustments were different among studies, most studies applied models with adjustment for BMI and in most studies an increased overall fracture risk in T2D compared to the reference population was found, with Hazard ratios varying from 1.2 to 2.4 (Bonds et al., 2006; Holmberg et al., 2006; Oei et al., 2013), including 3 meta-analyses (Wang et al., 2019; Moayeri et al., 2017; Janghorbani et al., 2007). Some of these studies reported no increased overall fracture risk in T2D, but the number of patients with T2D included in these latter studies was rather small (Ivers et al., 2001; Gerdhem et al., 2005). Several studies focussed on one specific fracture site, in particular hip fractures (Janghorbani et al., 2006; Nicodemus and Folsom, 2001; Ottenbacher et al., 2002; Lipscombe et al., 2007; Koh et al., 2010) and vertebral fractures (Yamamoto et al., 2009; Kilpadi et al., 2014; Napoli et al., 2018) and reported an increased fracture risk in T2D based on adjusted analyses (Janghorbani et al., 2006; Nicodemus and Folsom, 2001; Ottenbacher et al., 2002; Lipscombe et al., 2007; Koh et al., 2010; Yamamoto et al., 2009; Napoli et al., 2018). A few studies evaluated other fracture sites besides hip and vertebrae, but results were inconsistent possibly due to different study designs (Bonds et al., 2006; Schwartz et al., 2001; Zhang et al., 2019; Wang et al., 2019; Moayeri et al., 2017; Vestergaard, 2007; Vestergaard et al., 2005). Our adjusted analyses are quite in line with earlier studies since we also found in these analyses higher IRs for several fracture sites in patient with T2D as compared to the reference population. However, the findings of our crude analyses, pointing at a lower crude incidence of clavicle, radius/ulna and vertebral fractures in T2D and a higher crude incidence of ankle, foot, humerus and tibia/fibula fractures, are not in line with the majority of studies. In conclusion, our data stress the importance of reporting crude incidence or absolute risk data as these can better reflect absolute (or relative) fracture risks in T2D patients, and might better reflect the experienced risk in clinical practice, e.g. BMI adjusted analyses could falsely lead to the conclusion that a patient with T2D has an elevated fracture risk.

The proportion of patients with obesity was, as expected, significantly higher in our T2D patients compared to the reference population (54.8 % in T2D and 20.5 % in the reference population, Table 1). We observed significantly higher IRRs for hip, pelvis and rib fractures after adjustment for BMI in our T2D patients, as compared to crude IRRs, suggesting a protective effect of a higher BMI in T2D for these fractures. In contrast, a higher BMI seemed to have a negative effect on foot and ankle fractures. Previous studies suggested that a higher BMI might be protective for fractures at sites such as the hip and wrist (Ishii et al., 2014; Bouxsein et al., 2007) while it is a risk factor for fractures at other sites such as the lower extremities and the humerus (Lespessailles et al., 2019). It has been hypothesized that this is related to soft tissue padding at the hip and an altered fall mechanism (falling backwards or sidewards instead of forwards) (Mignardot et al., 2010; Prieto-Alhambra et al., 2012). Simultaneously, this altered fall mechanism in combination with a higher impact force during a fall may increase fracture risk at other skeletal sites, such as the lower extremities and the humerus (Lespessailles et al., 2019).

A protective effect of BMI in T2D, reflected by the lower crude IRRs, might especially be present in patient with a relatively short diabetes duration, when the impact of microvascular complications and the impairment of bone quality is less prominent. We included patients with newly treated T2D based on the first ever prescription of a NIAD and studied the fracture incidence during a mean follow-up of 7.7 years. This contrasts to most previous studies that examined fracture risk in patients with T2D in cohorts with a variable and mostly longer duration of disease. In these studies, it was reported that fracture incidence increased with a longer duration of T2D, especially for hip, proximal humerus and osteoporotic fractures, which was often not the case in patients with a shorter T2D duration (Schwartz et al., 2001; Ivers et al., 2001; Lipscombe et al., 2007; Koh et al., 2010; Leslie et al., 2007; Forsen et al., 1999; Melton et al., 2008). However, our adjusted analyses do suggest that also in T2D patients with a short duration of disease fracture risk can be increased, possibly due factors that impact on bone quality such as long-term elevated glucose levels, low-grade inflammation and oxidative stress (Bucala and Vlassara, 1995), which can be compensated by the ‘protective’ effects of an elevated BMI.

The crude incidence of all fractures was found to be marginally lower in patients with newly treated T2D although we consider this not a clinically relevant effect size. The findings in the total cohort were mainly determined by the findings in women, since we found no difference in the crude and adjusted IRRs of all fractures and most fracture sites in men. This suggests that BMI is a more important factor in women than in men with T2D regarding fracture incidence and risk. Our crude results concerning sex-specific IRs are in line with two previous studies that reported an increased risk of ankle and humerus fractures but not of wrist fractures in women with T2D and a decreased risk of all fractures in women but not in men with T2D. However one other study reported an increased risk of hip fractures in women but not in men with T2D and did not find a difference in non-vertebral fracture risk between men or women with or without T2D (Ahmed et al., 2006). Future detailed studies on the impact of BMI, glycaemia and the complications of T2D on bone morphology and quality, as well as the interaction with sex, might help to further unravel the underlying pathology.

Our study had various strengths and limitations. We were able to use the CPRD GOLD database, resulting in a large study population, which made it possible to study the incidence of multiple fracture sites in T2D. Second, we included all patients at start of NIAD treatment, which allowed us to study the pattern of fractures in newly drug treated patients with T2D, with a mean duration of disease less than one year at the start of this treatment. Third, we excluded all patients who used insulin before index date or were <30 years of age, prohibiting accidentally inclusion of T1D. Lastly, we included both women and man and stratified our fracture IRs by sex, most previous studies focussed on fracture risk in women with T2D.

An important limitation is that we had no data on BMD or HbA1c, important factors that might affect the adjusted IRRs. Second, we only included patients with T2D and a first NIAD prescription, without insulin use at or before index date, which might induce selection bias. Patients with T2D were not necessarily ‘newly diagnosed patients with T2D’ at the moment of their first NIAD prescription, since they might have developed or were even diagnosed with T2D a while before the start of NIADs. In addition, patients with T2D who are treated with diet only may have a different fracture pattern than those who receive oral glucose lowering medication. Although there are studies that focus on prediabetes (de Liefde et al., 2005; Strotmeyer ES et al., 2005), to our knowledge currently no study focussed on the subgroup of newly treated patients in relation to fracture pattern. Patients with T2D who are treated immediately with insulin (with or without a NIAD) often have a more severe status of the disease, that might be associated with a higher fracture risk (Hsu et al., 2018). In addition, we did not account for changes in diabetes medication during the study, including initiation of insulin treatment. Third, the reference population could have undiagnosed diabetes which would lead to an underestimation of our results. Fourth, the incidence of vertebral fractures could be underestimated since we only studied clinical symptomatic vertebral fractures based on Read codes. Fifth, due to the use of Read codes we have no data about the origin of fractures (spontaneous or traumatic). Sixth, we only determined BMI at baseline, changes over time were not taken into account.

In conclusion, crude fracture incidence of all fractures was marginally lower in patients with newly treated T2D during a mean follow-up of 7.7 years compared to the matched reference population. Crude fracture incidence differed according to fracture site, with a higher incidence of fractures at the lower extremities and the humerus in T2D and lower incidence of major osteoporotic and clavicle fractures. After adjustment for BMI an opposite pattern was seen, IRs for most fracture sites were significantly higher in the T2D cohort, in particular in women. Hence, BMI adjusted fracture incidence rate and risk estimates may be higher in T2D compared to the reference population while in contrast the crude fracture incidence, and therefore probably the absolute fracture risk, are at the same time actually lower compared to the reference population. This implies that BMI may have a protective impact on major osteoporotic fractures in patients with newly treated T2D, especially in women.

## Ethical approval

This research was in accordance with the principles of the Helsinki declaration and its later amendments or comparable ethical standards. Our study protocol (Protocol 18_275R) was approved by the Independent Scientific Advisory Committee (ISAC) for Medicines and Healthcare products Regulatory Agency (MHRA) database research. This study was a retrospective study and all data on patients are stored anonymously in CPRD. For this type of study formal consent is not required.

## Funding

This research was funded by 10.13039/501100004191Novo Nordisk grant (number NNF18OC0052064) sponsored by the Novo Nordisk Foundation.

## Ethics approval

This research was in accordance with the principles of the Helsinki declaration and its later amendments or comparable ethical standards. Our study protocol (Protocol 18_275R) was approved by the Independent Scientific Advisory Committee (ISAC) for Medicines and Healthcare products Regulatory Agency (MHRA) database research.

## Consent to participate

This study was a retrospective study and all data on patients are stored anonymously in the Clinical Practice Research Datalink (CPRD). For this type of study formal consent is not required.

## Consent for publication

This study was a retrospective study and all data on patients are stored anonymously in the Clinical Practice Research Datalink (CPRD). For this type of study formal consent is not required.

## Availability of data and material

Data that was obtained from the Clinical Practice Research Datalink (CPRD) GOLD is available for on-site audit purposes to qualified auditors, subject to further discussion and contractual agreements with the licensor of CPRD GOLD data.

## Code availability

SAS code that was used for data management and statistical analysis is available for on-site audit purposes to qualified auditors, subject to further discussion.

## CRediT authorship contribution statement

1. Design of the research and interpretation of data: all authors.
2. Data analysis: JD.
3. Original writing: CS.
4. Critical editing of written text and approval of the definitive version of the manuscript: all authors.
5. Agreement to be accountable for all aspects of the manuscript: PS, CS.

## Declaration of competing interest

The authors declare the following financial interests/personal relationships which may be considered as potential competing interests: Joop van den Bergh is involved in research that is sponsored by Amgen, Eli Lilly and UCB.

Peter Vestergaard is head of research in the Steno Diabetes Center North Jutland sponsored by the Novo Nordisk Foundation. The other authors, Cindy Sarodnik, Nicklas Rasmussen, Sandrine Bours, Nicolaas Schaper, Patrick Souverein, Morten Jensen, and Johanna Driessen, declare that they have no conflict of interest.

## Appendix A Supplementary data

Supplementary tables Image 1

Read Codes fractures Image 2
