# Supplementary material for: The incidence of fractures at various sites in newly treated patients with type 2 diabetes mellitus
Source: Bone Rep. 2022 Aug 22;17:101614. doi: 10.1016/j.bonr.2022.101614 (PMC9437792; doi:10.1016/j.bonr.2022.101614)
Supplement: Supplementary file 1 — Supplementary tables [file mmc1.docx]

**Title**

The incidence of fractures at various sites in newly treated subjects with Type 2 Diabetes Mellitus

**Journal name**

Bone Reports

**Authors**

Cindy Sarodnik^1^, Nicklas H. Rasmussen^2^, Sandrine P.G. Bours^3,4^, Nicolaas C. Schaper^1,3,4^, Peter Vestergaard^5^, Patrick C. Souverein^6^, Morten H. Jensen^2^, Johanna H.M. Driessen^1,6,7,8^, Joop P.W. van den Bergh^1,3,9^

**Affiliations**

1. NUTRIM Research School, Maastricht University, Maastricht, The Netherlands; 2. Steno Diabetes Center North Jutland, Aalborg University Hospital, Aalborg, Denmark; 3. Department of Internal Medicine, Maastricht University Medical Centre+; 4. CAPHRI Research School, Maastricht University, Maastricht, The Netherlands; 5. Steno Diabetes Center North Jutland, Department of Endocrinology, Aalborg University Hospital, Aalborg, Denmark; 6. Division of Pharmacoepidemiology & Clinical Pharmacology, Utrecht Institute for Pharmaceutical Sciences, Utrecht University, Utrecht, the Netherlands; 7. Department of Clinical Pharmacy and Toxicology, Maastricht University Medical Centre+, Maastricht, The Netherlands  8. CARIM Research School, Maastricht University, Maastricht, The Netherlands; 9. Department of Internal Medicine, VieCuri Medical Center, Venlo, the Netherlands.

**Corresponding author**

Patrick Souverein

Division of Pharmacoepidemiology and Clinical Pharmacology

Utrecht Institute for Pharmaceutical Sciences, Utrecht University

PO BOX 80082, 3508 TB, Utrecht, the Netherlands

E-mail address: p.c.souverein@uu.nl

*Caption Figures (These figures were created in Microsoft PowerPoint or Excel)*

**Table S.1 The incidence rate ratios for different fracture sites in T2D compared to matched reference population**

| ***Fracture site*** | **IRR crude (CI 95%)** | **IRR model 1 (CI 95%)** | **IRR model 2 (CI 95%)** |  | **IRR crude/**  **IRR model 1 (CI 95%)** | **IRR crude/**  **IRR model 2 (CI 95%)** |
| --- | --- | --- | --- | --- | --- | --- |
| All | 0.97 (0.94-0.99) | 1.07 (1.04-1.10)* | 1.06 (1.03-1.10)* |  | 1.11 (1.09-1.12)* | 1.10 (1.08-1.11)* |
| Hip | 0.96 (0.89-1.03) | 1.44 (1.33-1.55)* | 1.39 (1.29-1.51)* |  | 1.50 (1.45-1.55)* | 1.46 (1.41-1.51)* |
| Humerus | 1.11 (1.03-1.20)* | 1.20 (1.10-1.30)* | 1.18 (1.09-1.29)* |  | 1.08 (1.04-1.12)* | 1.06 (1.03-1.10)* |
| Pelvis | 0.89 (0.78-1.03) | 1.23 (1.05-1.44)* | 1.23 (1.05-1.44)* |  | 1.37 (1.29-1.46)* | 1.38 (1.29-1.47)* |
| Ribs | 1.08 (0.95-1.22) | 1.25 (1.09-1.42)* | 1.23 (1.08-1.41)* |  | 1.16 (1.10-1.22)* | 1.14 (1.08-1.21)* |
| Tibia/Fibula | 1.17 (1.03-1.33)* | 1.19 (1.03-1.37)* | 1.20 (1.04-1.38)* |  | 1.02 (0.96-1.08) | 1.03 (0.96-1.10) |
|  |  |  |  |  |  |  |
| Radius/Ulna | 0.81 (0.75-0.86)* | 0.88 (0.82-0.95)* | 0.87 (0.81-0.94)* |  | 1.09 (1.06-1.12)* | 1.08 (1.05-1.12)* |
|  |  |  |  |  |  |  |
| Ankle | 1.16 (1.06-1.28)* | 1.02 (0.92-1.13) | 1.03 (0.92-1.14) |  | 0.88 (0.84-0.92)* | 0.88 (0.84-0.92)* |
| Carpal | 1.00 (0.92-1.08) | 1.01 (0.92-1.10) | 1.02 (0.93-1.12) |  | 1.01 (0.97-1.05) | 1.02 (0.98-1.06) |
| Clavicle | 0.67 (0.56-0.81)* | 0.85 (0.70-1.03) | 0.85 (0.70-1.04) |  | 1.26 (1.17-1.35)* | 1.26 (1.17-1.36)* |
| Femur | 0.89 (0.74-1.07) | 1.20 (0.98-1.48) | 1.17 (0.95-1.45) |  | 1.35 (1.23-1.49)* | 1.32 (1.19-1.46)* |
| Foot | 1.11 (1.01-1.22)* | 1.04 (0.94-1.16) | 1.06 (0.95-1.17) |  | 0.94 (0.90-0.98)* | 0.96 (0.91-1.00) |
| Patella | 0.96 (0.74-1.26) | 1.21 (0.90-1.61) | 1.24 (0.92-1.66) |  | 1.25 (1.11-1.41)* | 1.28 (1.13-1.45)* |
| Scapula | 1.01 (0.78-1.31) | 0.98 (0.74-1.31) | 1.00 (0.75-1.33) |  | 0.97 (0.87-1.09) | 0.99 (0.87-1.12) |
| Skull | 1.00 (0.83-1.19) | 1.18 (0.97-1.44) | 1.19 (0.97-1.46) |  | 1.18 (1.09-1.29)* | 1.20 (1.10-1.31)* |
| Vertebrae | 0.83 (0.75-0.92)* | 1.00 (0.89-1.12) | 0.99 (0.88-1.11) |  | 1.20 (1.15-1.26)* | 1.19 (1.14-1.25)* |

Model 1 is adjusted for BMI; Model 2; Model 1 + additionally adjusted for smoking status, alcohol use, the history of any fracture at index date, the history of diagnosis of osteoporosis at index date or a prescription for anti-osteoporosis medication in the 6 months prior to the index date*. CI* confidence interval, *BMI* body mass index, *IRR* incidence rate ratio, * statistically significant results.

**Table S.2A** **The incidence rate ratios for different fracture sites in women with T2D versus the matched reference population.**

| ***Fracture site*** | **IRR crude (CI 95%)** | **IRR model 1 (CI 95%)** | **IRR model 2 (CI 95%)** |  | **IRR crude/model 1 (CI 95%)** | **IRR crude/model 2 (CI 95%)** |
| --- | --- | --- | --- | --- | --- | --- |
| All | 0.95 (0.91-0.98)* | 1.13 (1.09-1.18)* | 1.13 (1.08-1.17)* |  | 1.19 (1.17-1.22)* | 1.19 (1.17-1.21)* |
| Hip | 1.00 (0.92-1.09) | 1.66 (1.51-1.83)* | 1.63 (1.48-1.79)* |  | 1.66 (1.59-1.73)* | 1.62 (1.55-1.70)* |
| Humerus | 1.11 (1.01-1.21)* | 1.26 (1.14-1.39)* | 1.25 (1.13-1.39)* |  | 1.14 (1.09-1.19)* | 1.13 (1.08-1.19)* |
| Patella | 1.10 (0.78-1.54) | 1.54 (1.06-2.23)* | 1.60 (1.10-2.32)* |  | 1.40 (1.19-1.66)* | 1.45 (1.23-1.71)* |
| Pelvis | 0.93 (0.79-1.10) | 1.42 (1.19-1.71)* | 1.43 (1.19-1.72)* |  | 1.53 (1.42-1.65)* | 1.54 (1.42-1.67)* |
| Ribs | 0.99 (0.81-1.19) | 1.25 (1.01-1.55)* | 1.23 (0.99-1.52) |  | 1.27 (1.16-1.40)* | 1.24 (1.13-1.37)* |
| Tibia/Fibula | 1.14 (0.97-1.35) | 1.24 (1.03-1.49)* | 1.24 (1.03-1.49)* |  | 1.09 (1.00-1.18) | 1.09 (1.00-1.18) |
|  |  |  |  |  |  |  |
| Ankle | 1.15 (1.02-1.30)* | 1.04 (0.91-1.19) | 1.05 (0.91-1.20) |  | 0.91 (0.86-0.96)* | 0.91 (0.86-0.97)* |
| Carpal | 1.01 (0.90-1.12) | 1.07 (0.94-1.21) | 1.08 (0.95-1.22) |  | 1.06 (1.01-1.12)* | 1.07 (1.01-1.13)* |
| Clavicle | 0.66 (0.50-0.87)* | 0.91 (0.67-1.24) | 0.90 (0.66-1.23) |  | 1.38 (1.22-1.57)* | 1.37 (1.20-1.57)* |
| Femur | 0.86 (0.69-1.08) | 1.29 (0.99-1.66) | 1.27 (0.98-1.64) |  | 1.49 (1.32-1.69)* | 1.47 (1.30-1.67)* |
| Foot | 1.07 (0.95-1.21) | 1.05 (0.92-1.20) | 1.07 (0.94-1.22) |  | 0.98 (0.93-1.04) | 1.00 (0.94-1.06) |
| Radius/Ulna | 0.77 (0.72-0.84)* | 0.92 (0.84-1.00) | 0.93 (0.85-1.01) |  | 1.19 (1.15-1.24)* | 1.19 (1.15-1.24)* |
| Scapula | 1.01 (0.70-1.45) | 1.02 (0.69-1.51) | 1.04 (0.70-1.55) |  | 1.01 (0.86-1.18) | 1.03 (0.87-1.22) |
| Skull | 0.92 (0.70-1.19) | 1.18 (0.88-1.58) | 1.19 (0.88-1.61) |  | 1.29 (1.13-1.46)* | 1.30 (1.14-1.48)* |
| Vertebrae | 0.82 (0.72-0.93)* | 1.00 (0.89-1.12) | 1.06 (0.91-1.23) |  | 1.29 (1.21-1.37)* | 1.30 (1.22-1.39)* |

Model 1 is adjusted for BMI; Model 2; Model 1 + additionally adjusted for smoking status, alcohol use, the history of any fracture at index date, the history of diagnosis of osteoporosis at index date or a prescription for anti-osteoporosis medication in the 6 months prior to the index date. *CI* confidence interval, *BMI* body mass index, *IRR* incidence rate ratio, * statistically significant results.

**Table S.2B** **The incidence rate ratios for different fracture sites in men with T2D versus the matched reference population.**

| ***Fracture site*** | **IRR crude (CI 95%)** | **IRR model 1 (CI 95%)** | **IRR model 2 (CI 95%)** |  | **IRR crude/model 1 (CI 95%)** | **IRR crude/model 2 (CI 95%)** |
| --- | --- | --- | --- | --- | --- | --- |
| Hip | 0.88 (0.78-0.99)* | 1.18 (1.03-1.35)* | 1.11 (0.97-1.27) |  | 1.34 (1.27-1.41)* | 1.26 (1.19-1.33)* |
| Humerus | 1.15 (1.00-1.32)* | 1.21 (1.03-1.41)* | 1.18 (1.01-1.38)* |  | 1.05 (0.99-1.12) | 1.03 (0.96-1.10) |
| Ribs | 1.14 (0.98-1.34) | 1.23 (1.04-1.46)* | 1.21 (1.02-1.44)* |  | 1.08 (1.01-1.15)* | 1.06 (0.99-1.14) |
|  |  |  |  |  |  |  |
| All | 1.01 (0.97-1.06) | 1.05 (1.00-1.10) | 1.04 (0.99-1.09) |  | 1.04 (1.02-1.06)* | 1.03 (1.01-1.05)* |
| Ankle | 1.20 (1.03-1.40) | 1.06 (0.89-1.24) | 1.06 (0.90-1.26) |  | 0.88 (0.82-0.94)* | 0.89 (0.83-0.95)* |
| Carpal | 0.99 (0.88-1.12) | 0.97 (0.85-1.11) | 1.00 (0.87-1.14) |  | 0.98 (0.93-1.03) | 1.01 (0.95-1.06) |
| Clavicle | 0.68 (0.54-0.87) | 0.80 (0.62-1.03) | 0.81 (0.63-1.04) |  | 1.17 (1.07-1.28)* | 1.18 (1.07-1.30)* |
| Femur | 0.96 (0.70-1.32) | 1.13 (0.78-1.65) | 1.07 (0.73-1.56) |  | 1.18 (1.01-1.38)* | 1.11 (0.94-1.32) |
| Foot | 1.18 (1.01-1.38) | 1.10 (0.93-1.30) | 1.13 (0.96-1.34) |  | 0.93 (0.87-1.00)* | 0.96 (0.89-1.03) |
| Patella | 0.79 (0.51-1.22) | 0.89 (0.56-1.42) | 0.89 (0.56-1.42) |  | 1.13 (0.95-1.33) | 1.13 (0.94-1.35) |
| Pelvis | 0.83 (0.63-1.08) | 0.98 (0.73-1.31) | 0.95 (0.70-1.29) |  | 1.18 (1.05-1.33)* | 1.16 (1.02-1.31)* |
| Radius/Ulna | 0.93 (0.81-1.07) | 0.90 (0.78-1.04) | 0.89 (0.77-1.03) |  | 0.97 (0.92-1.03) | 0.96 (0.90-1.01) |
| Scapula | 1.01 (0.69-1.48) | 0.97 (0.64-1.48) | 0.97 (0.64-1.47) |  | 0.96 (0.81-1.14) | 0.96 (0.80-1.14) |
| Skull | 1.07 (0.84-1.36) | 1.18 (0.90-1.54) | 1.21 (0.92-1.59) |  | 1.10 (0.99-1.23) | 1.13 (1.01-1.27)* |
| Tibia/Fibula | 1.22 (1.00-1.48) | 1.16 (0.94-1.45) | 1.20 (0.96-1.50) |  | 0.96 (0.87-1.05) | 0.99 (0.90-1.09) |
| Vertebrae | 0.85 (0.73-1.00) | 1.00 (0.89-1.12) | 0.93 (0.78-1.12) |  | 1.15 (1.08-1.23)* | 1.09 (1.02-1.17)* |

Model 1 is adjusted for BMI; Model 2; Model 1 + additionally adjusted for smoking status, alcohol use, the history of any fracture at index date, the history of diagnosis of osteoporosis at index date or a prescription for anti-osteoporosis medication in the 6 months prior to the index date*. CI* confidence interval, *BMI* body mass index, *IRR* incidence rate ratio, * statistically significant results.
