# Supplementary material for: The incidence of fractures at various sites in newly treated patients with type 2 diabetes mellitus
Source: Bone Rep. 2022 Aug 22;17:101614. doi: 10.1016/j.bonr.2022.101614 (PMC9437792; doi:10.1016/j.bonr.2022.101614)
Supplement: Supplementary file 2 — Read Codes fractures [file mmc2.pdf]

icd_gr_term	readcode	readterm
Ankle	S34..00	Fracture of ankle
Ankle	S344.12	Pott's fracture - ankle
Ankle	S34x.00	Closed fracture ankle, unspecified
Ankle	S348.00	Fracture of medial malleolus
Ankle	7K1L800	Closed reduction of fracture of ankle
Ankle	S340.00	Closed fracture ankle, medial malleolus
Ankle	S349.00	Fracture of lateral malleolus
Ankle	S342000	Closed fracture ankle, lateral malleolus, low
Ankle	S344.00	Closed fracture ankle, bimalleolar
Ankle	S342.00	Closed fracture ankle, lateral malleolus
Ankle	S34z.00	Fracture of ankle, NOS
Ankle	S347.00	Open fracture ankle, trimalleolar
Ankle	S346.00	Closed fracture ankle, trimalleolar
Ankle	S344.11	Dupuytren's fracture, fibula
Ankle	S4G..00	Fracture-dislocation or subluxation ankle
Ankle	S343.00	Open fracture ankle, lateral malleolus
Ankle	S345.00	Open fracture ankle, bimalleolar
Ankle	S4G3.00	Open fracture-subluxation, ankle joint
Ankle	S341.00	Open fracture ankle, medial malleolus
Ankle	S4G0.00	Closed fracture-dislocation, ankle joint
Ankle	S342100	Closed fracture ankle, lateral malleolus, high
Ankle	S34y.00	Open fracture ankle, unspecified
Ankle	S344000	Closed fracture ankle, bimalleolar, low fibular fracture
Ankle	S343000	Open fracture ankle, lateral malleolus, low
Ankle	S347000	Open fracture ankle, trimalleolar, low fibular fracture
Ankle	S346100	Closed fracture ankle, trimalleolar, high fibular fracture
Ankle	S344100	Closed fracture ankle, bimalleolar, high fibular fracture
Ankle	S346000	Closed fracture ankle, trimalleolar, low fibular fracture
Ankle	S4G1.00	Open fracture-dislocation, ankle joint
Ankle	S4G2.00	Closed fracture-subluxation, ankle joint
Ankle	S345000	Open fracture ankle, bimalleolar, low fibular fracture
Ankle	S343100	Open fracture ankle, lateral malleolus, high
Carpal	S26..00	Fracture of one or more phalanges of hand
Carpal	S26..12	Thumb fracture excluding base
Carpal	S242000	Fracture of scaphoid
Carpal	S242200	Fracture of other metacarpal bone
Carpal	S25..11	Hand fracture - metacarpal bone
Carpal	S25..00	Fracture of metacarpal bone
Carpal	S26z.00	Fracture of one or more phalanges of hand NOS
Carpal	S242300	Multiple fractures of metacarpal bones
Carpal	S26..11	Finger fracture
Carpal	S240400	Closed fracture pisiform
Carpal	7K1LM00	Closed reduction of fracture of wrist
Carpal	S240100	Closed fracture of the scaphoid
Carpal	S263.00	Fracture of other finger
Carpal	S250.00	Closed fracture of metacarpal bone(s)
Carpal	S262.00	Fracture of thumb

Carpal	S242100	Fracture of first metacarpal bone
Carpal	S242.00	Fracture at wrist and hand level
Carpal	S264.00	Multiple fractures of fingers
Carpal	S24..11	Hand fracture - carpal bone
Carpal	S4D..00	Fracture-dislocation/subluxation finger/thumb
Carpal	S240200	Closed fracture lunate
Carpal	S250100	Cls # thumb metacarpal base, intra-articular, Bennett
Carpal	S2B..00	Fracture of bone of hand
Carpal	S240.00	Closed fracture of carpal bone
Carpal	S240A00	Closed fracture scaphoid, proximal pole
Carpal	S241100	Open fracture of the scaphoid
Carpal	S240700	Closed fracture capitate
Carpal	S4C2.00	Closed fracture-subluxation of the wrist
Carpal	S240B00	Closed fracture scaphoid, waist, transverse
Carpal	S24..00	Fracture of carpal bone
Carpal	S260D00	Closed fracture finger proximal phalanx
Carpal	S240500	Closed fracture trapezium
Carpal	S4C0300	Closed fracture-dislocation, carpometacarpal joint
Carpal	S240300	Closed fracture triquetral
Carpal	S240E00	Closed fracture scaphoid, tuberosity
Carpal	S240800	Closed fracture hamate
Carpal	S260K00	Closed fracture finger middle phalanx
Carpal	S261D00	Open fracture finger proximal phalanx
Carpal	S240z00	Closed fracture of carpal bone NOS
Carpal	S260100	Clsd # mid/prox phalanx/phalanges, unspecified part
Carpal	S240600	Closed fracture trapezoid
Carpal	S4C0100	Closed fracture-dislocation radiocarpal joint
Carpal	S240900	Closed fracture hamate, hook
Carpal	S4C1.00	Open fracture dislocation wrist
Carpal	S24z.00	Fracture of carpal bone NOS
Carpal	S241400	Open fracture pisiform
Carpal	S240D00	Closed fracture scaphoid, waist, comminuted
Carpal	S4C3100	Open fracture-subluxation radiocarpal joint
Carpal	S240C00	Closed fracture scaphoid, waist, oblique
Carpal	S4C1100	Open fracture-dislocation radiocarpal joint
Carpal	S241E00	Open fracture scaphoid, tuberosity
Carpal	S4C2100	Closed fracture-subluxation radiocarpal joint
Carpal	S241300	Open fracture triquetral
Carpal	S4C0600	Closed fracture-dislocation peri-lunate trans-scaphoid
Carpal	S4C2400	Closed fracture-subluxation lunate (volar)
Carpal	S241A00	Open fracture scaphoid, proximal pole
Carpal	S241500	Open fracture trapezium
Carpal	S240y00	Closed fracture of other carpal bone
Carpal	S240000	Closed fracture of carpal bone, unspecified
Carpal	S4C3.00	Open fracture-subluxation of the wrist
Carpal	S241C00	Open fracture scaphoid, waist, oblique
Carpal	S4C1600	Open fracture-dislocation peri-lunate trans-scaphoid
Carpal	S4C2600	Closed fracture-subluxation peri-lunate trans-scaphoid

Carpal	S4C0400	Closed fracture-dislocation lunate (volar)
Carpal	S241D00	Open fracture scaphoid, waist, comminuted
Carpal	S241700	Open fracture capitate
Carpal	S241.00	Open fracture of carpal bone
Carpal	S4C0200	Closed fracture-dislocation mid carpal
Carpal	S241000	Open fracture of carpal bone, unspecified
Carpal	S4C3600	Open fracture-subluxation peri-lunate trans-scaphoid
Carpal	S4C2300	Closed fracture-subluxation, carpometacarpal joint
Carpal	S241200	Open fracture lunate
Carpal	S241800	Open fracture hamate
Carpal	S240F00	Closed fracture carpal bones, multiple
Carpal	S241B00	Open fracture scaphoid, waist, transverse
Carpal	S4C3300	Open fracture-subluxation, carpometacarpal joint
Carpal	S4C0500	Closed fracture-dislocation peri-lunate (dorsal)
Carpal	S4C1300	Open fracture-dislocation carpometacarpal joint
Clavicle	S20..00	Fracture of clavicle
Clavicle	S20..11	Collar bone fracture
Clavicle	S292.00	Multiple fractures of clavicle, scapula and humerus
Clavicle	S201200	Open fracture clavicle, shaft
Clavicle	S20z.00	Fracture of clavicle NOS
Clavicle	S200z00	Closed fracture of clavicle NOS
Clavicle	S200200	Closed fracture clavicle, shaft
Clavicle	S200300	Closed fracture clavicle, lateral end
Clavicle	S200.00	Closed fracture of clavicle
Clavicle	S200100	Closed fracture clavicle, medial end
Clavicle	S200000	Closed fracture of clavicle, unspecified part
Clavicle	S201.00	Open fracture of clavicle
Clavicle	S292100	Open multiple fractures of clavicle, scapula and humerus
Clavicle	S201300	Open fracture clavicle, lateral end
Clavicle	S201000	Open fracture of clavicle, unspecified part
Clavicle	S201z00	Open fracture of clavicle NOS
Femur unspe	S31z.00	Fracture of femur, NOS
Femur unspe	S312300	Closed fracture distal femur, supracondylar
Femur unspe	S312100	Closed fracture of femoral condyle, unspecified
Femur unspe	S310.00	Closed fracture of femur, shaft or unspecified part
Femur unspe	S31..00	Other fracture of femur
Femur unspe	S315.00	Fracture of lower end of femur
Femur unspe	S314.00	Fracture of shaft of femur
Femur unspe	S311100	Open fracture shaft of femur
Femur unspe	S310011	Thigh fracture NOS
Femur unspe	S310012	Upper leg fracture NOS
Femur unspe	S3x2.00	Multiple fractures of femur
Femur unspe	S312200	Closed fracture of femur, lower epiphysis
Femur unspe	S312.11	Closed fracture of femur, distal end
Femur unspe	S310100	Closed fracture shaft of femur
Femur unspe	S312.00	Closed fracture distal femur
Femur unspe	S313.11	Open fracture of femur, distal end
Femur unspe	S311000	Open fracture of femur, unspecified part

Femur unspe	S313500	Open fracture distal femur, lateral condyle
Femur unspe	S310000	Closed fracture of femur, unspecified part
Femur unspe	S312500	Closed fracture distal femur, lateral condyle
Femur unspe	7K1G200	Primary open reduction+external fixation of femoral fracture
Femur unspe	S313300	Open fracture distal femur, supracondylar
Femur unspe	S311.00	Open fracture of femur, shaft or unspecified part
Femur unspe	S313000	Open fracture distal femur, unspecified
Femur unspe	S312400	Closed fracture distal femur, medial condyle
Femur unspe	S313200	Open fracture of femur, lower epiphysis
Femur unspe	S313100	Open fracture of femoral condyle, unspecified
Femur unspe	S313.00	Open fracture distal femur
Femur unspe	S310z00	Closed fracture of shaft or unspecified part, NOS
Femur unspe	S312000	Closed fracture of distal femur, unspecified
Femur unspe	S312600	Closed fracture distal femur, bicondylar (T-Y fracture)
Femur unspe	S312x00	Closed fracture distal femur, comminuted/intra-articular
Femur unspe	S312z00	Closed fracture of distal femur not otherwise specified
Femur unspe	S313400	Open fracture distal femur, medial condyle
Femur unspe	Syu7200	[X]Fractures of other parts of femur
Femur unspe	S313x00	Open fracture distal femur, comminuted/intra-articular
Femur unspe	S313z00	Open fracture of distal femur not otherwise specified
Femur unspe	S311z00	Open fracture of femur, shaft or unspecified part, NOS
Femur unspe	S301A00	Open fracture of femur, upper epiphysis
Foot	S35..11	Metatarsal bone fracture
Foot	S35..00	Fracture of one or more tarsal and metatarsal bones
Foot	S352.11	March fracture
Foot	S354.00	Fracture of calcaneus
Foot	S36..11	Toe fracture
Foot	S362.00	Fracture of great toe
Foot	S355.00	Fracture of talus
Foot	S36..00	Fracture of one or more phalanges of foot
Foot	S35..12	Tarsal bone fracture
Foot	S352700	Closed fracture metatarsal
Foot	S360.00	Closed fracture of one or more phalanges of foot
Foot	S352300	Closed fracture cuboid
Foot	S356.00	Fracture of metatarsal bone
Foot	S363.00	Fracture of other toe
Foot	7K1LA00	Closed reduction of fracture of toe
Foot	S350.11	Heel bone fracture
Foot	S350.00	Closed fracture of calcaneus
Foot	S3x4.00	Multiple fractures of foot
Foot	7K1LB00	Closed reduction of fracture of hallux
Foot	S352200	Closed fracture navicular
Foot	S3xz.00	Other, multiple and ill-defined fractures of lower limb NOS
Foot	S352100	Closed fracture of talus
Foot	S350.12	Os calcis fracture
Foot	S352.00	Closed fracture of other tarsal and metatarsal bones
Foot	S4H..00	Fracture-dislocation or subluxation foot
Foot	S4H2300	Closed #-subluxation, metatarsophalangeal joint, single

Foot	S352000	Closed fracture of tarsal bone, unspecified
Foot	S352B00	Closed fracture metatarsal base
Foot	S360000	Closed fracture proximal phalanx, toe
Foot	S352C00	Closed fracture metatarsal shaft
Foot	S360200	Closed fracture distal phalanx, toe
Foot	S352F00	Closed fracture metatarsal, multiple
Foot	S362000	Closed fracture of great toe
Foot	S4H0400	Closed fracture-dislocation, IPJ, single toe
Foot	S352J00	Closed fracture of base of fifth metatarsal
Foot	S352D00	Closed fracture metatarsal neck
Foot	S360100	Closed fracture middle phalanx, toe
Foot	S352111	Closed fracture of astragalus
Foot	S362100	Open fracture of great toe
Foot	S36z.00	Fracture of one or more phalanges of foot NOS
Foot	S350100	Closed fracture calcaneus, intra-articular
Foot	S352400	Closed fracture medial cuneiform
Foot	S352E00	Closed fracture metatarsal head
Foot	S352H00	Closed fracture of cuneiforms
Foot	S352800	Closed fracture talus, head
Foot	S361.00	Open fracture of one or more phalanges of foot
Foot	7K1L900	Closed reduction of fracture of metatarsus
Foot	S35z.00	Fracture of tarsal and metatarsal bones NOS
Foot	S353100	Open fracture of talus
Foot	S4H1300	Open fracture-dislocation, metatarsophalangeal joint, single
Foot	S4H1000	Open fracture-dislocation, subtalar joint
Foot	S353H00	Open fracture cuneiforms
Foot	S353200	Open fracture navicular
Foot	S4H0300	Closed #-dislocation, metatarsophalangeal joint, single
Foot	S4H1400	Open fracture-dislocation, IPJ, single toe
Foot	S352z00	Closed fracture of one or more tarsal + metatarsal bones NOS
Foot	S351.00	Open fracture of calcaneus
Foot	S4H0.00	Closed fracture-dislocation foot
Foot	S4H2.00	Closed fracture-subluxation, foot
Foot	S3x..00	Other, multiple and ill-defined fractures of lower limb
Foot	S352A00	Closed fracture talus, body
Foot	S352900	Closed fracture talus, neck
Foot	S353z00	Open fracture of tarsal and metatarsal bones NOS
Foot	S353300	Open fracture cuboid
Foot	S352600	Closed fracture lateral cuneiform
Foot	S4H0500	Closed #-dislocation, metatarsophalangeal joint, multiple
Foot	S361000	Open fracture proximal phalanx, toe
Foot	S361200	Open fracture distal phalanx, toe
Foot	S4H1.00	Open fracture-dislocation, foot
Foot	S360300	Closed fracture multiple phalanges, toe
Foot	S353700	Open fracture metatarsal
Foot	S4H0000	Closed fracture-dislocation, subtalar joint
Foot	S4H2500	Closed #-subluxation, metatarsophalangeal joint, multiple
Foot	S350000	Closed fracture calcaneus, extra-articular

Foot	S352500	Closed fracture intermediate cuneiform
Foot	S361300	Open fracture multiple phalanges, toe
Foot	S352G00	Closed tarsal fractures, multiple
Foot	S353C00	Open fracture metatarsal shaft
Foot	S4H2100	Closed fracture-subluxation, midtarsal joint
Foot	S4H1500	Open #-dislocation, metatarsophalangeal joint, multiple
Foot	S4H3300	Open fracture-subluxation, metatarsophalangeal joint, single
Foot	S353F00	Open fracture metatarsal, multiple
Foot	S353400	Open fracture medial cuneiform
Foot	S353.00	Open fracture of other tarsal and metatarsal bones
Foot	S353J00	Open fracture of base of fifth metatarsal
Foot	S4H0200	Closed fracture-dislocation, tarsometatarsal joint
Foot	S4H3400	Open fracture-subluxation, IPJ, single toe
Foot	S361100	Open fracture middle phalanx, toe
Foot	S351100	Open fractures calcaneus, intra-articular
Foot	S4H0100	Closed fracture-dislocation, midtarsal joint
Foot	S4H2000	Closed fracture-subluxation, subtalar joint
Foot	S353B00	Open fracture metatarsal base
Foot	S4H2600	Closed fracture-subluxation, IPJ, multiple toes
Foot	S4H2200	Closed fracture-subluxation, tarsometatarsal joint
Foot	S4H1200	Open fracture-dislocation, tarsometatarsal joint
Foot	S4H0600	Closed fracture-dislocation, IPJ, multiple toes
Foot	S353900	Open fracture talus, neck
Foot	S4H3.00	Open fracture-subluxation, foot
Foot	S4H2400	Closed fracture-subluxation, IPJ, single toe
Foot	S353500	Open fracture intermediate cuneiform
Foot	Syu9400	[X]Fracture of other tarsal bones
Foot	S353A00	Open fracture talus, body
Foot	S353D00	Open fracture metatarsal neck
Hip	S30..11	Hip fracture
Hip	S30..00	Fracture of neck of femur
Hip	S302.00	Closed fracture of proximal femur, pertrochanteric
Hip	7K1L400	Closed reduction of fracture of hip
Hip	S302400	Closed fracture of femur, intertrochanteric
Hip	S30y.11	Hip fracture NOS
Hip	S300500	Cls # prox femur, subcapital, Garden grade unspec.
Hip	S30y.00	Closed fracture of neck of femur NOS
Hip	7K1L500	Closed reduction of fracture of femur
Hip	S302000	Cls # proximal femur, trochanteric section, unspecified
Hip	S302011	Closed fracture of femur, greater trochanter
Hip	S301800	Open fracture proximal femur,subcapital, Garden grade III
Hip	S30w.00	Closed fracture of unspecified proximal femur
Hip	S4E..00	Fracture-dislocation or subluxation hip
Hip	S304.00	Pertrochanteric fracture
Hip	S302200	Closed fracture proximal femur, subtrochanteric
Hip	S300700	Closed fracture proximal femur, subcapital, Garden grade II
Hip	S300900	Closed fracture proximal femur, subcapital, Garden grade IV
Hip	S300600	Closed fracture proximal femur, subcapital, Garden grade I

Hip	S300400	Closed fracture head of femur
Hip	S300800	Closed fracture proximal femur, subcapital, Garden grade III
Hip	S30z.00	Open fracture of neck of femur NOS
Hip	S300.00	Closed fracture proximal femur, transcervical
Hip	S301500	Open fracture proximal femur,subcapital, Garden grade unspec
Hip	S303400	Open fracture of femur, intertrochanteric
Hip	S300000	Cls # prox femur, intracapsular section, unspecified
Hip	S4E0.00	Closed fracture-dislocation, hip joint
Hip	S302z00	Cls # of proximal femur, pertrochanteric section, NOS
Hip	S302100	Closed fracture proximal femur, intertrochanteric, two part
Hip	S300A00	Closed fracture of femur, upper epiphysis
Hip	S302012	Closed fracture of femur, lesser trochanter
Hip	S300y00	Closed fracture proximal femur, other transcervical
Hip	S301000	Opn # proximal femur, intracapsular section, unspecified
Hip	S302300	Cls # proximal femur, intertrochanteric, comminuted
Hip	S300311	Closed fracture, base of neck of femur
Hip	S301900	Open fracture proximal femur,subcapital, Garden grade IV
Hip	S300300	Closed fracture proximal femur, basicervical
Hip	S30x.00	Open fracture of unspecified proximal femur
Hip	S4E1.00	Open fracture-dislocation, hip joint
Hip	S301600	Open fracture proximal femur,subcapital, Garden grade I
Hip	S303.00	Open fracture of proximal femur, pertrochanteric
Hip	S300z00	Closed fracture proximal femur, transcervical, NOS
Hip	S300200	Closed fracture proximal femur, midcervical section
Hip	S301700	Open fracture proximal femur,subcapital, Garden grade II
Hip	S300y11	Closed fracture of femur, subcapital
Hip	S301y00	Open fracture proximal femur, other transcervical
Hip	S300100	Closed fracture proximal femur, transepiphyseal
Hip	S303z00	Open fracture of proximal femur, pertrochanteric, NOS
Hip	S303200	Open fracture proximal femur, subtrochanteric
Hip	S301100	Open fracture proximal femur, transepiphyseal
Hip	S301400	Open fracture head, femur
Hip	S301y11	Open fracture of femur, subcapital
Hip	S301.00	Open fracture proximal femur, transcervical
Hip	S4E2.00	Closed fracture-subluxation, hip joint
Hip	S303011	Open fracture of femur, greater trochanter
Humerus	S22..00	Fracture of humerus
Humerus	S28..11	Ill-defined fracture of arm
Humerus	S224.11	Elbow fracture - closed
Humerus	S228.00	Fracture of lower end of humerus
Humerus	S226.00	Fracture of upper end of humerus
Humerus	S28z.00	Ill-defined fractures of upper limb NOS
Humerus	S2...11	Arm fracture
Humerus	S2...00	Fracture of upper limb
Humerus	7K1LF00	Closed reduction of fracture of humerus
Humerus	S224100	Closed fracture distal humerus, supracondylar
Humerus	S225.11	Elbow fracture - open
Humerus	S4A0.00	Closed fracture-dislocation shoulder

Humerus	S224600	Closed fracture distal humerus, lateral epicondyle
Humerus	S293.00	Multiple fractures of forearm
Humerus	S221.00	Open fracture of the proximal humerus
Humerus	S4B0000	Closed fracture-dislocation elbow joint
Humerus	S4B..00	Fracture-dislocation or subluxation elbow
Humerus	S22z.00	Fracture of humerus NOS
Humerus	S221.11	Shoulder fracture - open
Humerus	S220300	Closed fracture proximal humerus, greater tuberosity
Humerus	S220.00	Closed fracture of the proximal humerus
Humerus	S220100	Closed fracture proximal humerus, neck
Humerus	S224.00	Closed fracture of the distal humerus
Humerus	S225700	Open fracture distal humerus, medial epicondyle
Humerus	S224200	Closed fracture distal humerus, lateral condyle
Humerus	S222000	Closed fracture of humerus NOS
Humerus	S4A2100	Closed fracture-subluxation acromio-clavicular joint
Humerus	S4A0100	Closed fracture-dislocation acromio-clavicular joint
Humerus	S222100	Closed fracture of humerus, shaft
Humerus	S224800	Closed fracture distal humerus, capitellum
Humerus	S224700	Closed fracture distal humerus, medial epicondyle
Humerus	S220400	Closed fracture proximal humerus, head
Humerus	S220700	Closed fracture proximal humerus, four part
Humerus	S2z..00	Fracture of upper limb NOS
Humerus	S227.00	Fracture of shaft of humerus
Humerus	S225100	Open fracture distal humerus, supracondylar
Humerus	S224400	Closed fracture of distal humerus, condyle(s) unspecified
Humerus	S225800	Open fracture distal humerus, capitellum
Humerus	S220200	Closed fracture of proximal humerus, anatomical neck
Humerus	S224z00	Closed fracture of distal humerus, not otherwise specified
Humerus	S223.00	Open fracture of humerus, shaft or unspecified part
Humerus	S4B0.00	Closed fracture-dislocation elbow
Humerus	S4A0000	Closed fracture-dislocation shoulder joint
Humerus	S224000	Closed fracture of elbow, unspecified part
Humerus	S225.00	Open fracture of the distal humerus
Humerus	S4A2.00	Closed fracture-subluxation shoulder
Humerus	S222.00	Closed fracture of humerus, shaft or unspecified part
Humerus	S220z00	Closed fracture of proximal humerus not otherwise specified
Humerus	S220600	Closed fracture proximal humerus, three part
Humerus	S223100	Open fracture of humerus, shaft
Humerus	S224300	Closed fracture distal humerus, medial condyle
Humerus	S4B1.00	Open fracture-dislocation elbow
Humerus	S225000	Open fracture of elbow, unspecified part
Humerus	S220000	Closed fracture of proximal humerus, unspecified part
Humerus	S221z00	Open fracture of proximal humerus not otherwise specified
Humerus	S225z00	Open fracture of distal humerus, not otherwise specified
Humerus	S225600	Open fracture distal humerus, lateral epicondyle
Humerus	S221300	Open fracture proximal humerus, greater tuberosity
Humerus	S223000	Open fracture of humerus NOS
Humerus	S4A1100	Open fracture-dislocation acromio-clavicular joint

Humerus	S224500	Closed fracture of distal humerus, trochlea
Humerus	S220500	Closed fracture of humerus, upper epiphysis
Humerus	S221100	Open fracture proximal humerus, neck
Humerus	S224900	Closed fracture distal humerus, bicondylar (T-Y fracture)
Humerus	S221000	Open fracture of proximal humerus, unspecified part
Humerus	S4B1000	Open fracture-dislocation elbow joint
Humerus	S4B2.00	Closed fracture-subluxation elbow
Humerus	S221500	Open fracture of humerus, upper epiphysis
Humerus	S225200	Open fracture distal humerus, lateral condyle
Humerus	S4A2000	Closed fracture-subluxation shoulder joint
Humerus	S4A3100	Open fracture-subluxation acromio-clavicular joint
Humerus	S222z00	Closed fracture of humerus, shaft or unspecified part NOS
Humerus	S224x00	Closed fracture of distal humerus, multiple
Humerus	S4B0100	Closed fracture-dislocation superior radio-ulnar joint
Humerus	S225500	Open fracture of distal humerus, trochlea
Humerus	S4A1.00	Open fracture-dislocation shoulder
Humerus	S4B2000	Closed fracture-subluxation elbow joint
Humerus	S225300	Open fracture distal humerus, medial condyle
Humerus	S221400	Open fracture proximal humerus, head
Humerus	S221600	Open fracture proximal humerus, three part
Humerus	S221700	Open fracture proximal humerus, four part
Humerus	S221200	Open fracture of proximal humerus, anatomical neck
Humerus	S223z00	Open fracture of humerus, shaft or unspecified part NOS
Humerus	S4B3.00	Open fracture-subluxation elbow
Humerus	S225400	Open fracture of distal humerus, condyle(s) unspecified
Humerus	S225900	Open fracture distal humerus, bicondylar (T-Y fracture)
Patella	S32..00	Fracture of patella
Patella	S32..11	#Knee-cap
Patella	7K1F500	Primary open reduction fracture patella fixat tension band
Patella	S321.00	Open fracture of the patella
Patella	S4F4.00	Closed fracture-dislocation, patello-femoral joint
Patella	S320.00	Closed fracture of the patella
Patella	S321200	Open fracture patella, distal pole
Patella	S32z.00	Fracture of patella, NOS
Patella	S4F7.00	Open fracture-subluxation, patello-femoral joint
Patella	S4F6.00	Closed fracture-subluxation, patello-femoral joint
Patella	S320400	Closed fracture patella, comminuted (stellate)
Patella	S320200	Closed fracture patella, distal pole
Patella	S320000	Closed fracture patella, transverse
Patella	S321000	Open fracture patella, transverse
Patella	S321400	Open fracture patella, comminuted (stellate)
Patella	S320100	Closed fracture patella, proximal pole
Patella	S320300	Closed fracture patella, vertical
Patella	S4F5.00	Open fracture-dislocation, patello-femoral joint
Pelvis	S13..00	Fracture or disruption of pelvis
Pelvis	S130.00	Closed fracture acetabulum
Pelvis	S10B500	Fracture of pubis
Pelvis	S132.00	Closed fracture pubis

Pelvis	S132100	Closed fracture pelvis, multiple pubic rami - stable
Pelvis	S132000	Closed fracture pelvis, single pubic ramus
Pelvis	S10B400	Fracture of acetabulum
Pelvis	S134z00	Other or multiple closed fracture of pelvis NOS
Pelvis	S108.00	Closed fracture pelvis, coccyx
Pelvis	S134600	Closed fracture pelvis, iliac wing
Pelvis	S10B300	Fracture of ilium
Pelvis	S134400	Closed fracture pelvis, anterior superior iliac spine
Pelvis	S13y.00	Closed fracture of pelvis NOS
Pelvis	S132z00	Closed fracture pubis NOS
Pelvis	S134.00	Other or multiple closed fracture of pelvis
Pelvis	S134800	Closed fracture dislocation of sacro-iliac joint
Pelvis	S4J2100	Closed fracture-subluxation of pelvis
Pelvis	S133000	Open fracture pelvis, single pubic ramus
Pelvis	S134100	Closed fracture pelvis, ischium
Pelvis	S4J0100	Closed fracture-dislocation of pelvis
Pelvis	S135z00	Other/multiple open fracture of pelvis NOS
Pelvis	S132y00	Other specified closed fracture pubis
Pelvis	S134500	Closed fracture pelvis, anterior inferior iliac spine
Pelvis	S134000	Closed fracture of ilium, unspecified
Pelvis	S134300	Closed fracture pelvis, ischial tuberosity
Pelvis	S135400	Open fracture pelvis, anterior superior iliac spine
Pelvis	S4J1100	Open fracture-dislocation of pelvis
Pelvis	S130z00	Closed fracture acetabulum NOS
Pelvis	S132200	Closed fracture pelvis, multiple pubic rami - unstable
Pelvis	S133.00	Open fracture of pubis
Pelvis	S133100	Open fracture pelvis, multiple pubic rami - stable
Pelvis	S134700	Closed vertical fracture of ilium
Pelvis	S131.00	Open fracture acetabulum
Pelvis	S130300	Closed fracture acetabulum, posterior column
Pelvis	S130y00	Other specified closed fracture acetabulum
Pelvis	S131y00	Other specified open fracture acetabulum
Pelvis	S13z.00	Open fracture of pelvis NOS
Pelvis	S131z00	Open fracture acetabulum NOS
Pelvis	S135.00	Other or multiple open fracture of pelvis
Pelvis	S4J3100	Open fracture-subluxation of pelvis
Pelvis	S135600	Open fracture pelvis, iliac wing
Pelvis	S135300	Open fracture pelvis, ischial tuberosity
Pelvis	S130400	Closed fracture acetabulum, floor
Pelvis	S133z00	Open fracture of pubis NOS
Pelvis	S130000	Closed fracture acetabulum, anterior lip alone
Pelvis	S130200	Closed fracture acetabulum, anterior column
Pelvis	S133y00	Other specified open fracture of pubis
Pelvis	S130100	Closed fracture acetabulum, posterior lip alone
Pelvis	S135800	Open fracture dislocation of sacro-iliac joint
Pelvis	S135000	Open fracture of ilium, unspecified
Rad ulna	S23x111	Fracture of radius NOS
Rad ulna	S23B.00	Fracture of lower end of radius

Rad ulna	S234.11	Wrist fracture - closed
Rad ulna	S234100	Closed Colles' fracture
Rad ulna	S23z.00	Fracture of radius and ulna, NOS
Rad ulna	S23x211	Fracture of ulna NOS
Rad ulna	S234200	Closed fracture of the distal radius, unspecified
Rad ulna	S237.00	Fracture of upper end of radius
Rad ulna	S230300	Closed Monteggia's fracture
Rad ulna	S234700	Closed Smith's fracture
Rad ulna	S233.00	Open fracture of radius and ulna, shaft
Rad ulna	S23x300	Closed fracture of the radius and ulna
Rad ulna	S235100	Open Colles' fracture
Rad ulna	S23C.00	Fracture of lower end of both ulna and radius
Rad ulna	S235B00	Open fracture radial styloid
Rad ulna	S23..00	Fracture of radius and ulna
Rad ulna	S234B00	Closed fracture radial styloid
Rad ulna	7K1LL00	Closed reduction of fracture of radius and or ulna
Rad ulna	S230600	Closed fracture radius, head
Rad ulna	7K1LE00	Closed reduction of fracture of elbow
Rad ulna	S231600	Open fracture radial head
Rad ulna	S230700	Closed fracture radius, neck
Rad ulna	S239.00	Fracture of shaft of radius
Rad ulna	S238.00	Fracture of shaft of ulna
Rad ulna	S231B00	Open fracture olecranon, intra-articular
Rad ulna	S23y300	Open fracture of the radius and ulna
Rad ulna	S234300	Closed fracture of ulna, styloid process
Rad ulna	S4C1000	Open fracture-dislocation, distal radio-ulnar joint
Rad ulna	S230100	Closed fracture olecranon, extra-articular
Rad ulna	S235.11	Wrist fracture - open
Rad ulna	S23A.00	Fracture of shafts of both ulna and radius
Rad ulna	S231300	Open Monteggia's fracture
Rad ulna	S23..11	Forearm fracture
Rad ulna	S234900	Closed volar Barton's fracture
Rad ulna	S235300	Open fracture of ulna, styloid process
Rad ulna	S230B00	Closed fracture olecranon, intra-articular
Rad ulna	S23x.00	Closed fracture of radius and ulna, unspecified part
Rad ulna	S230200	Closed fracture of ulna, coronoid
Rad ulna	S4C0000	Closed fracture-dislocation distal radio-ulnar joint
Rad ulna	S23x100	Closed fracture of radius (alone), unspecified
Rad ulna	S234.00	Closed fracture of radius and ulna, lower end
Rad ulna	S234000	Closed fracture of forearm, lower end, unspecified
Rad ulna	S4C..00	Fracture-dislocation or subluxation of wrist
Rad ulna	S234D00	Closed fracture distal radius, extra-articular, other type
Rad ulna	S23x200	Closed fracture of ulna (alone), unspecified
Rad ulna	S232.00	Closed fracture of radius and ulna, shaft
Rad ulna	S235.00	Open fracture of radius and ulna, lower end
Rad ulna	S234z00	Closed fracture of forearm, lower end, NOS
Rad ulna	S233z00	Open fracture of radius and ulna, shaft, NOS
Rad ulna	S234E00	Closed fracture distal radius, intra-articular, other type

Rad ulna	S234600	Closed fracture radius and ulna, distal
Rad ulna	S23y200	Open fracture of ulna (alone), unspecified
Rad ulna	S231100	Open fracture olecranon, extra-articular
Rad ulna	S235800	Open Galeazzi fracture
Rad ulna	S236.00	Fracture of upper end of ulna
Rad ulna	S232z00	Closed fracture of radius and ulna, shaft, NOS
Rad ulna	S230900	Closed fracture of the proximal radius
Rad ulna	S230000	Closed fracture of proximal forearm, unspecified part
Rad ulna	S230711	Closed # radius neck
Rad ulna	S23y100	Open fracture of radius (alone), unspecified
Rad ulna	S230800	Closed fracture proximal radius, comminuted
Rad ulna	S230400	Closed fracture of proximal ulna, comminuted
Rad ulna	S230500	Closed fracture of the proximal ulna
Rad ulna	S4C0.00	Closed fracture dislocation of wrist
Rad ulna	S235700	Open Smith's fracture
Rad ulna	S231700	Open fracture radial neck
Rad ulna	S232300	Closed fracture radius and ulna, middle
Rad ulna	S23xz00	Closed fracture of radius and ulna, NOS
Rad ulna	S231A00	Open fracture radius and ulna, proximal
Rad ulna	S235600	Open fracture radius and ulna, distal
Rad ulna	S234800	Closed Galeazzi fracture
Rad ulna	S234500	Closed fracture distal ulna, unspecified
Rad ulna	S234400	Closed fracture of ulna, lower epiphysis
Rad ulna	S232100	Closed fracture of the radial shaft
Rad ulna	S230z00	Closed fracture of proximal forearm not otherwise specified
Rad ulna	S230.00	Closed fracture of proximal radius and ulna
Rad ulna	S230A00	Closed fracture radius and ulna, proximal
Rad ulna	S4C2000	Closed fracture-subluxation, distal radio-ulnar jt
Rad ulna	S232200	Closed fracture of the ulnar shaft
Rad ulna	S234C00	Closed fracture distal radius, intra-articular, die-punch
Rad ulna	S235200	Open fracture of the distal radius, unspecified
Rad ulna	S231.00	Open fracture of proximal radius and ulna
Rad ulna	Q203111	Birth fracture of radius
Rad ulna	S233300	Open fracture radius and ulna, middle
Rad ulna	S235500	Open fracture distal ulna - other
Rad ulna	S234A00	Closd dorsal Barton's fracture
Rad ulna	S231000	Open fracture of proximal forearm, unspecified
Rad ulna	S23x000	Closed fracture of forearm, unspecified
Rad ulna	S232000	Closed fracture of radius, shaft, unspecified
Rad ulna	S234111	Smith's fracture - closed
Rad ulna	S231200	Open fracture of ulna, coronoid
Rad ulna	S234911	Closed volar Barton's fracture-dislocation
Rad ulna	S235D00	Open fracture distal radius, extra-articular other type
Rad ulna	S23y.00	Open fracture of radius and ulna, unspecified part
Rad ulna	S231z00	Open fracture of forearm, upper end, NOS
Rad ulna	S234A11	Closed dorsal Barton's fracture-dislocation
Rad ulna	S4C3000	Open fracture-subluxation, distal radio-ulnar joint
Rad ulna	S233000	Open fracture of radius, shaft, unspecified

Rad ulna	S235000	Open fracture of forearm, lower end, unspecified
Rad ulna	S231500	Open fracture of the proximal ulna
Rad ulna	S235900	Open volar Barton's fracture
Rad ulna	S235E00	Open fracture distal radius, intra-articular other type
Rad ulna	S231900	Open fracture of the proximal radius
Rad ulna	S23yz00	Open fracture of radius and ulna, NOS
Rad ulna	S234912	Closed volar Barton fracture-subluxation
Rad ulna	S233100	Open fracture of the radial shaft
Rad ulna	S235z00	Open fracture of forearm, lower end, NOS
Rad ulna	S233200	Open fracture of the ulnar shaft
Rad ulna	S23y000	Open fracture of forearm, unspecified
Rad ulna	S231800	Open fracture proximal radius, comminuted
Rad ulna	Q203112	Birth fracture of ulna
Rad ulna	S235400	Open fracture of ulna, lower epiphysis
Ribs	S120.00	Closed fracture rib
Ribs	S122.00	Closed fracture sternum
Ribs	S120000	Closed fracture of rib, unspecified
Ribs	S120A00	Cough fracture
Ribs	S127.00	Fracture of rib
Ribs	S127000	Multiple fractures of ribs
Ribs	S120900	Closed fracture multiple ribs
Ribs	S12z.11	Rib fracture NOS
Ribs	S128.00	Fracture of sternum
Ribs	S120100	Closed fracture of one rib
Ribs	S29..12	Multiple rib fractures
Ribs	S4J1200	Open fracture-dislocation sterno-clavicular joint, anterior
Ribs	S1z..00	Fracture of neck and trunk NOS
Ribs	S12z.12	Sternum fracture NOS
Ribs	S120z00	Closed fracture of rib(s) NOS
Ribs	S127100	Cough fracture of ribs
Ribs	S125200	Closed fracture of thyroid cartilage
Ribs	S120400	Closed fracture of four ribs
Ribs	S125100	Closed fracture of hyoid bone
Ribs	S125000	Closed fracture larynx
Ribs	S120200	Closed fracture of two ribs
Ribs	S121200	Open fracture of two ribs
Ribs	S12z.00	Fracture of rib(s), sternum, larynx or trachea NOS
Ribs	S121.00	Open fracture rib
Ribs	S4J0200	Closed #-dislocation sterno-clavicular joint, anterior
Ribs	S12X.00	Fracture of bony thorax, part unspecified
Ribs	S125300	Closed fracture of trachea
Ribs	S120500	Closed fracture of five ribs
Ribs	S120300	Closed fracture of three ribs
Ribs	S12..00	Fracture of rib(s), sternum, larynx and trachea
Ribs	S12X000	Closed fracture of bony thorax part unspecified
Ribs	S4J2200	Closed #-subluxation sterno-clavicular joint, anterior
Ribs	S4J2000	Closed fracture-subluxation of sternum
Ribs	S123.00	Open fracture sternum

Ribs	S4J0000	Closed fracture-dislocation of sternum
Ribs	S120600	Closed fracture of six ribs
Ribs	S120800	Closed fracture of eight or more ribs
Ribs	S121900	Open fracture multiple ribs
Ribs	S125.00	Closed fracture larynx and trachea
Ribs	S121000	Open fracture of rib, unspecified
Ribs	S121700	Open fracture of seven ribs
Ribs	S120700	Closed fracture of seven ribs
Ribs	S4J0300	Closed #-dislocation sterno-clavicular joint, posterior
Ribs	S126300	Open fracture of trachea
Ribs	S4J1000	Open fracture-dislocation of sternum
Ribs	S4J3000	Open fracture-subluxation of sternum
Ribs	S12y000	Closed fracture of other parts of bony thorax
Ribs	S126100	Open fracture of hyoid bone
Scapula	S21..00	Fracture of scapula
Scapula	S210300	Closed fracture scapula, glenoid
Scapula	S210400	Closed fracture scapula, blade
Scapula	7K1LG00	Closed reduction of fracture of shoulder
Scapula	S21..11	Shoulder blade fracture
Scapula	S210100	Closed fracture scapula, acromion
Scapula	S210.00	Closed fracture of scapula
Scapula	S21z.00	Fracture of scapula NOS
Scapula	S210200	Closed fracture scapula, coracoid
Scapula	S211.00	Open fracture of scapula
Scapula	S210600	Closed fracture scapula, neck
Scapula	S210000	Closed fracture of scapula, unspecified part
Scapula	S210500	Closed fracture scapula, spine
Scapula	S211100	Open fracture scapula, acromion
Scapula	S210z00	Closed fracture of scapula NOS
Scapula	S211300	Open fracture scapula, glenoid
Scapula	S211200	Open fracture scapula, coracoid
Scapula	S211600	Open fracture scapula, neck
Scapula	S211400	Open fracture scapula, blade
Scapula	S211000	Open fracture of scapula, unspecified part
Scapula	S211z00	Open fracture of scapula NOS
Skull	S020.00	Closed fracture nose
Skull	S0...00	Fracture of skull
Skull	S024100	Closed fracture zygoma
Skull	S01..00	Fracture of base of skull
Skull	S022.00	Fracture of mandible, closed
Skull	7J03100	Reduction of fracture of nasal bones NEC
Skull	S021.00	Open fracture nose
Skull	S00..11	Frontal bone fracture
Skull	S024000	Closed fracture maxilla
Skull	7J13400	Reduction of Le Fort 1 fracture of maxilla
Skull	7J03200	Reduction of fracture of zygomatic bones
Skull	S02x100	Fracture of orbit NOS, closed
Skull	S028000	Fracture of nasal bones

Skull	S01..19	Temporal bone fracture
Skull	7J12.00	Reduction of fracture of mandible
Skull	S00..12	Parietal bone fracture
Skull	S02..00	Fracture of face bones
Skull	S01..15	Occiput bone fracture
Skull	S020.11	Closed fracture nasal bone
Skull	S1...00	Fracture of neck and trunk
Skull	7206100	Open reduction of fracture of orbit
Skull	S028300	Fracture of mandible
Skull	7J12200	Closed reduction of fracture of mandible NEC
Skull	S022100	Closed fracture of mandible, condylar process
Skull	S025100	Open fracture zygoma
Skull	S024.00	Fracture of malar or maxillary bones, closed
Skull	S00..00	Fracture of vault of skull
Skull	7J03.00	Reduction of fracture of facial bone
Skull	S022.12	Fracture of lower jaw, closed
Skull	7J12.11	Reduction of fracture of jaw NEC
Skull	S02z.11	Jaw fracture NOS
Skull	S001000	Closed #skull vlt + intracranial injury, unspec state consc
Skull	S00z.00	Fracture of vault of skull NOS
Skull	7206400	Open reduction of fracture of orbit and internal fixation
Skull	S028100	Fracture of orbital floor
Skull	7J13.00	Reduction of fracture of maxilla
Skull	S03z.11	Depressed skull fracture NOS
Skull	S026.00	Closed orbital blow-out fracture
Skull	S02z.00	Fracture of facial bone NOS
Skull	7K1LD00	Closed reduction of fracture of nasal bone
Skull	S02xz00	Fracture of other facial bones, closed, NOS
Skull	S02x000	Fracture of alveolus, closed
Skull	7J12y00	Other specified reduction of fracture of mandible
Skull	S031.00	Closed fracture of skull NOS with intracranial injury
Skull	S001.00	Closed fracture vault of skull with intracranial injury
Skull	7J03z00	Reduction of fracture of facial bone NOS
Skull	S022400	Closed fracture of mandible, ramus, unspecified
Skull	7J12100	Open reduction of fracture of mandible NEC
Skull	S022000	Closed fracture mandible (site unspecified)
Skull	S02x.00	Closed fracture other facial bone
Skull	S028200	Fracture of malar and maxillary bones
Skull	7403600	Outfracture of turbinates of nose
Skull	S028.00	Fracture of skull and facial bones
Skull	7J13000	Reduction of fracture of alveolus of maxilla
Skull	S02y100	Fracture of orbit NOS, open
Skull	SR10.00	Fractures involving head with neck
Skull	S025000	Open fracture maxilla
Skull	S027.00	Open orbital blow-out fracture
Skull	S044.00	Multiple fractures involving skull and facial bones
Skull	S01z.00	Fracture of base of skull NOS
Skull	S0z..00	Fracture of skull NOS

Skull	S02yz00	Fracture of other facial bones,open, NOS
Skull	7J13200	Closed reduction of fracture of maxilla NEC
Skull	S022z00	Fracture of mandible, closed, NOS
Skull	S024.11	Fracture of upper jaw, closed
Skull	7K1Ez00	Prim open reduction fracture bone & extramedull fixation NOS
Skull	S023.11	Fracture of lower jaw, open
Skull	S021.11	Open fracture nasal bone
Skull	7J12z00	Reduction of fracture of mandible NOS
Skull	S01..16	Orbital roof fracture
Skull	S022x00	Closed fracture of mandible, multiple sites
Skull	S023.00	Fracture of mandible, open
Skull	S022500	Closed fracture of mandible, angle of jaw
Skull	S022200	Closed fracture of mandible, subcondylar
Skull	7J03300	Reduction of closed fracture of orbit bone
Skull	S011600	Closed #skull bse + intracranial injury, LOC unspec duration
Skull	S04..00	Multiple fractures involving skull or face with other bones
Skull	S000.00	Closed fracture vault of skull without intracranial injury
Skull	S025.00	Fracture of malar or maxillary bones, open
Skull	S024z00	Fracture of malar or maxillary bones, closed, NOS
Skull	S02y000	Fracture of alveolus, open
Skull	S04z.00	Multiple fractures involving skull/face with other bones NOS
Skull	S000200	Closed #skull vlt no intracranial injury, <1hr loss of consc
Skull	S041300	Closed #skull/face, mult + intracranial inj, 1-24hrs LOC
Skull	7J13500	Reduction of Le Fort 2 fracture of maxilla
Skull	S003.00	Open fracture vault of skull with intracranial injury
Skull	S000500	Closed #skull vlt no intracranial inj,>24hr LOC not restored
Skull	S023x00	Open fracture of mandible, multiple sites
Skull	S02x200	Fracture of palate, closed
Skull	S023500	Open fracture of mandible, angle of jaw
Skull	S022700	Closed fracture of mandible, alveolar border of body
Skull	S002.00	Open fracture vault of skull without intracranial injury
Skull	S03z.00	Skull fracture NOS
Skull	S030.00	Closed fracture of skull NOS without intracranial injury
Skull	S010000	Closed #skull bse no intracranial injury, unspec state consc
Skull	S010100	Closed #skull bse no intracranial injury, no loss of consc
Skull	S022800	Closed fracture of mandible, body, other and unspecified
Skull	S02y.00	Open fracture other facial bone
Skull	S022300	Closed fracture of mandible, coronoid process
Skull	S001z00	Closed #skull vlt with intracranial injury+concussion unspec
Skull	S023z00	Fracture of mandible, open, NOS
Skull	S023800	Open fracture of mandible, body, other and unspecified
Skull	7J13600	Reduction of Le Fort 3 fracture of maxilla
Skull	S023100	Open fracture of mandible, condylar process
Skull	S003600	Open #skull vlt + intracranial injury, LOC unspec duration
Skull	S03..00	Other and unqualified skull fractures
Skull	S022.11	Fracture of inferior maxilla, closed
Skull	S003z00	Open #skull vlt with intracranial injury + concussion unspec
Skull	S043000	Open #skull/face, mult + intracranial inj, unspec consc

Skull	S001600	Closed #skull vlt + intracranial injury, LOC unspec duration
Skull	S000100	Closed #skull vlt no intracranial injury, no loss of consc
Skull	S010.00	Closed fracture base of skull without intracranial injury
Skull	7J03y00	Other specified reduction of fracture of facial bone
Skull	S011.00	Closed fracture base of skull with intracranial injury
Skull	S003000	Open #skull vlt + intracranial injury, unspec state of consc
Skull	S031600	Closed #skull NOS + intracranial inj, LOC unspec duration
Skull	S032z00	Open #skull NOS no intracranial inj + concussion unspec
Skull	S000000	Closed #skull vlt no intracranial injury, unspec state consc
Skull	S002000	Open #skull vlt no intracranial injury, unspec state consc
Skull	S002100	Open #skull vlt no intracranial injury, no loss of consc
Skull	S030100	Closed #skull NOS no intracranial inj, no loss of consc
Skull	S000300	Closed #skull vlt no intracranial injury, 1-24hr loss consc
Skull	S033.00	Open fracture of skull NOS with intracranial injury
Skull	S031200	Closed #skull NOS + intracranial inj, <1hr loss of consc
Skull	S023400	Open fracture of mandible, ramus, unspecified
Skull	S025z00	Fracture of malar or maxillary bones, open, NOS
Skull	7J13z00	Reduction of fracture of maxilla NOS
Skull	S031300	Closed #skull NOS + intracranial inj, 1-24hrs loss of consc
Skull	S012.00	Open fracture base skull without mention intracranial injury
Skull	S025.11	Fracture of upper jaw, open
Skull	S023000	Open fracture mandible (site unspecified)
Skull	S022600	Closed fracture of mandible, symphysis of body
Skull	S011400	Closed #skull bse + intracranial injury, >24hr LOC+recovery
Skull	S041z00	Closed #skull/face,mult + intracran inj, concussion unspec
Skull	S040200	Closed #skull/face, mult, no intracranial inj, <1hr LOC
Skull	S001400	Closed #skull vlt + intracranial injury, >24hr LOC+recovery
Skull	S043400	Open #skull/face, mult + intracran inj, >24hr LOC + recovery
Skull	S041.00	Mult #skull/face+other bones, closed + intracranial injury
Skull	S042.00	Mult #skull/face + other bones, open, no intracranial injury
Skull	S000z00	Closed #skull vlt no intracranial injury + concussion unspec
Skull	S003100	Open #skull vlt + intracranial injury, no loss of consc
Skull	S013400	Open #skull bse + intracranial injury, >24hr LOC + recovery
Skull	SR10000	Closed fractures involving head with neck
Skull	7J13y00	Other specified reduction of fracture of maxilla
Skull	7403900	Surgical outfracture of turbinate of nose
Skull	S030z00	Closed #skull NOS no intracranial inj + concussion unspec
Skull	S040.00	Mult #skull/face+other bones, closed, no intracranial injury
Skull	S000600	Closed #skull vlt no intracranial inj, LOC unspec duration
Skull	S041000	Closed #skull/face, mult + intracranial inj, unspec consc
Skull	S001300	Closed #skull vlt + intracranial injury, 1-24hr loss consc
Skull	S032.00	Open #skull NOS without mention of intracranial injury
Skull	S010200	Closed #skull bse no intracranial injury, <1hr loss of consc
Skull	Syu0400	[X]Fracture of skull and facial bones, part unspecified
Skull	S010z00	Closed #skull bse no intracranial injury + concussion unspec
Skull	S041600	Closed #skull/face,mult + intracran inj, LOC unspec duration
Skull	S013.00	Open fracture base of skull with intracranial injury
Tib_Fib	S339.00	Fracture of fibula alone

Tib_Fib	S33x000	Closed fracture of tibia, unspecified part, NOS
Tib_Fib	S3...11	Leg fracture
Tib_Fib	S33..00	Fracture of tibia and fibula
Tib_Fib	S33x100	Closed fracture of fibula, unspecified part, NOS
Tib_Fib	S33x200	Closed fracture of tibia and fibula, unspecified part
Tib_Fib	7K1L700	Closed reduction of fracture of tibia and or fibula
Tib_Fib	S339000	Closed fracture of distal fibula
Tib_Fib	S336.00	Fracture of upper end of tibia
Tib_Fib	S337.00	Fracture of shaft of tibia
Tib_Fib	S334100	Closed fracture distal tibia, intra-articular
Tib_Fib	S3...00	Fracture of lower limb
Tib_Fib	S3x3.00	Multiple fractures of lower leg
Tib_Fib	S338.00	Fracture of lower end of tibia
Tib_Fib	S3X..00	Fracture of lower leg, part unspecified
Tib_Fib	S339100	Open fracture of distal fibula
Tib_Fib	S330300	Closed fracture proximal tibia, medial condyle (plateau)
Tib_Fib	S330400	Closed fracture proximal tibia, lateral condyle (plateau)
Tib_Fib	S330012	Closed fracture of tibial tuberosity
Tib_Fib	S33z.00	Fracture of tibia and fibula, NOS
Tib_Fib	S334.00	Closed fracture distal tibia
Tib_Fib	S335000	Open fracture distal tibia, extra-articular
Tib_Fib	S335.00	Open fracture distal tibia
Tib_Fib	S333.00	Open fracture of tibia/fibula, shaft
Tib_Fib	S333000	Open fracture shaft of tibia
Tib_Fib	S333z00	Open fracture of tibia and fibula, shaft, NOS
Tib_Fib	S33y.00	Open fracture of tibia and fibula, unspecified part, NOS
Tib_Fib	S33y100	Open fracture of fibula, unspecified part, NOS
Tib_Fib	S332100	Closed fracture shaft of fibula
Tib_Fib	S330000	Closed fracture of the proximal tibia
Tib_Fib	S4F1.00	Open fracture-dislocation, knee joint
Tib_Fib	S33y200	Open fracture of tibia and fibula, unspecified part
Tib_Fib	S33x.00	Closed fracture of tibia and fibula, unspecified part, NOS
Tib_Fib	S332.00	Closed fracture of tibia/fibula, shaft
Tib_Fib	S33y000	Open fracture of tibia, unspecified part, NOS
Tib_Fib	S4F..00	Fracture-dislocation or subluxation knee
Tib_Fib	S331100	Open fracture proximal fibula
Tib_Fib	S332200	Closed fracture of tibia and fibula, shaft
Tib_Fib	S330100	Closed fracture proximal fibula
Tib_Fib	S4F2.00	Closed fracture-subluxation, knee joint
Tib_Fib	S331000	Open fracture of the proximal tibia
Tib_Fib	S330600	Closed fracture spine, tibia
Tib_Fib	S332000	Closed fracture shaft of tibia
Tib_Fib	S334000	Closed fracture distal tibia, extra-articular
Tib_Fib	S33x.11	Lower leg fracture NOS
Tib_Fib	S330700	Closed fracture tubercle, tibia
Tib_Fib	S331.00	Open fracture of tibia and fibula, proximal
Tib_Fib	S330500	Closed fracture proximal tibia, bicondylar
Tib_Fib	S370.00	Closed fracture of lower limb, level unspecified

Tib_Fib	S4F0.00	Closed fracture-dislocation, knee joint
Tib_Fib	S33xz00	Closed fracture of tibia and fibula, unspecified part, NOS
Tib_Fib	S330z00	Closed fracture of tibia and fibula, proximal NOS
Tib_Fib	S371.00	Open fracture of lower limb, level unspecified
Tib_Fib	S331300	Open fracture proximal tibia, medial condyle (plateau)
Tib_Fib	S331400	Open fracture proximal tibia, lateral condyle (plateau)
Tib_Fib	S330.00	Closed fracture of tibia and fibula, proximal
Tib_Fib	S331700	Open fracture tubercle, tibia
Tib_Fib	S331012	Open fracture of tibial tuberosity
Tib_Fib	S333100	Open fracture shaft of fibula
Tib_Fib	S330900	Closed fracture fibula, neck
Tib_Fib	S330800	Closed fracture fibula, head
Tib_Fib	S330011	Closed fracture of tibial condyles
Tib_Fib	S331200	Open fracture of tibia and fibula, proximal
Tib_Fib	S330200	Closed fracture of tibia and fibula, proximal
Tib_Fib	S332z00	Closed fracture of tibia and fibula, shaft, NOS
Tib_Fib	S4F3.00	Open fracture-subluxation, knee joint
Tib_Fib	S331z00	Open fracture of tibia and fibula, proximal NOS
Tib_Fib	S33yz00	Open fracture of tibia and fibula, unspecified part, NOS
Tib_Fib	S331600	Open fracture spine, tibia
Tib_Fib	S335100	Open fracture distal tibia, intra-articular
Tib_Fib	S33A.00	Fracture of tibia
Tib_Fib	S331011	Open fracture of tibial condyles
Tib_Fib	Syu8300	[X]Fractures of other parts of lower leg
Vertebrae	S10B200	Fracture of coccyx
Vertebrae	N331.12	Collapse of vertebra NOS
Vertebrae	S10A.00	Fracture of neck
Vertebrae	S10x.00	Closed fracture of spine, unspecified,
Vertebrae	S10B100	Fracture of sacrum
Vertebrae	S104.00	Closed fracture lumbar vertebra
Vertebrae	N331L00	Collapse of vertebra due to osteoporosis NOS
Vertebrae	S10..12	Fracture of vertebra without spinal cord lesion
Vertebrae	S15..00	Fracture of thoracic vertebra
Vertebrae	N331J00	Collapse of lumbar vertebra due to osteoporosis
Vertebrae	S10..00	Fracture of spine without mention of spinal cord injury
Vertebrae	S104100	Closed fracture lumbar vertebra, wedge
Vertebrae	S10B600	Multiple fractures of lumbar spine and pelvis
Vertebrae	N331F00	Collapse of thoracic vertebra
Vertebrae	S10B000	Fracture of lumbar vertebra
Vertebrae	S150.00	Multiple fractures of thoracic spine
Vertebrae	S100.00	Closed fracture of cervical spine
Vertebrae	N331G00	Collapse of lumbar vertebra
Vertebrae	S102y00	Other specified closed fracture thoracic vertebra
Vertebrae	S10B.00	Fracture of lumbar spine and pelvis
Vertebrae	N331900	Osteoporosis + pathological fracture thoracic vertebrae
Vertebrae	S100000	Closed fracture of unspecified cervical vertebra
Vertebrae	N331011	Collapse of thoracic vertebra
Vertebrae	S106.00	Closed fracture sacrum

Vertebrae	S100200	Closed fracture axis
Vertebrae	N1y1.00	Fatigue fracture of vertebra
Vertebrae	N331E00	Collapse of cervical vertebra
Vertebrae	N331800	Osteoporosis + pathological fracture lumbar vertebrae
Vertebrae	N331K00	Collapse of thoracic vertebra due to osteoporosis
Vertebrae	S10A200	Multiple fractures of cervical spine
Vertebrae	7J43.00	Fixation of fracture of spine
Vertebrae	7J42.00	Other reduction of fracture of spine
Vertebrae	N331111	Collapse of lumbar vertebra
Vertebrae	S101500	Open fracture of fifth cervical vertebra
Vertebrae	S100700	Closed fracture of seventh cervical vertebra
Vertebrae	S102.00	Closed fracture thoracic vertebra
Vertebrae	S100500	Closed fracture of fifth cervical vertebra
Vertebrae	S100600	Closed fracture of sixth cervical vertebra
Vertebrae	S10A000	Fracture of first cervical vertebra
Vertebrae	S102100	Closed fracture thoracic vertebra, wedge
Vertebrae	N331.11	Collapse of spine NOS
Vertebrae	S104400	Closed fracture lumbar vertebra, transverse process
Vertebrae	S10..11	Fracture of transverse process spine - no spinal cord lesion
Vertebrae	N331100	Pathological fracture of lumbar vertebra
Vertebrae	N331000	Pathological fracture of thoracic vertebra
Vertebrae	S11x.00	Closed fracture of spine with spinal cord lesion unspecified
Vertebrae	7J41.00	Decompression of fracture of spine
Vertebrae	S11..00	Fracture of spine with spinal cord lesion
Vertebrae	S100611	C6 vertebra closed fracture without spinal cord lesion
Vertebrae	S100211	C2 vertebra closed fracture without spinal cord lesion
Vertebrae	S10z.00	Fracture of spine without mention of spinal cord lesion NOS
Vertebrae	S10A100	Fracture of second cervical vertebra
Vertebrae	7J42100	Open reduction of fracture of spine NEC
Vertebrae	S100511	C5 vertebra closed fracture without spinal cord lesion
Vertebrae	S104300	Closed fracture lumbar vertebra, spinous process
Vertebrae	S100711	C7 vertebra closed fracture without spinal cord lesion
Vertebrae	N331D00	Collapsed vertebra NOS
Vertebrae	S102000	Closed fracture thoracic vertebra, burst
Vertebrae	S100A00	Closed fracture axis, odontoid process
Vertebrae	S102z00	Closed fracture thoracic vertebra not otherwise specified
Vertebrae	S100400	Closed fracture of fourth cervical vertebra
Vertebrae	S100z00	Closed fracture of cervical spine not otherwise specified
Vertebrae	S100C00	Closed fracture axis, spinous process
Vertebrae	S100111	C1 vertebra closed fracture - no spinal cord lesion
Vertebrae	S105.00	Open fracture lumbar vertebra
Vertebrae	S104000	Closed fracture lumbar vertebra, burst
Vertebrae	S11..12	Fracture of vertebra with spinal cord lesion
Vertebrae	N331.14	Osteoporotic vertebral collapse
Vertebrae	N331H00	Collapse of cervical vertebra due to osteoporosis
Vertebrae	7J42.11	Other reduction of fracture of spine and stabilisation
Vertebrae	N331A00	Osteoporosis + pathological fracture cervical vertebrae
Vertebrae	S102400	Closed fracture thoracic vertebra, transverse process

Vertebrae	S100311	C3 vertebra closed fracture without spinal cord lesion
Vertebrae	S100H00	Closed fracture cervical vertebra, wedge
Vertebrae	S101200	Open fracture axis
Vertebrae	S100K00	Closed fracture cervical vertebra, spinous process
Vertebrae	S109.00	Open fracture pelvis, coccyx
Vertebrae	S100x00	Multiple closed fractures of cervical vertebrae
Vertebrae	S101.00	Open fracture of cervical spine
Vertebrae	S116.00	Closed fracture of sacrum with spinal cord lesion
Vertebrae	S101711	C7 vertebra open fracture without spinal cord lesion
Vertebrae	S101311	C3 vertebra open fracture without spinal cord lesion
Vertebrae	S100300	Closed fracture of third cervical vertebra
Vertebrae	S104200	Closed fracture lumbar vertebra, spondylolysis
Vertebrae	S103100	Open fracture thoracic vertebra, wedge
Vertebrae	S101611	C6 vertebra open fracture without spinal cord lesion
Vertebrae	S103.00	Open fracture thoracic vertebra
Vertebrae	S100L00	Closed fracture cervical vertebra, transverse process
Vertebrae	S102300	Closed fracture thoracic vertebra, spinous process
Vertebrae	S101600	Open fracture of sixth cervical vertebra
Vertebrae	S105100	Open fracture lumbar vertebra, wedge
Vertebrae	S10y.00	Open fracture of spine, unspecified,
Vertebrae	S150100	Open multiple fracture of thoracic spine
Vertebrae	S107.00	Open fracture sacrum
Vertebrae	S100411	C4 vertebra closed fracture without spinal cord lesion
Vertebrae	S100G00	Closed fracture cervical vertebra, burst
Vertebrae	S101111	C1 vertebra open fracture without spinal cord lesion
Vertebrae	S101100	Open fracture atlas
Vertebrae	S100900	Closed fracture atlas, comminuted
Vertebrae	S11..11	Fracture of transverse process of spine + spinal cord lesion
Vertebrae	S106000	Closed compression fracture sacrum
Vertebrae	S106100	Closed vertical fracture of sacrum
Vertebrae	S101x00	Multiple open fractures of cervical vertebrae
Vertebrae	S105000	Open fracture lumbar vertebra, burst
Vertebrae	S100D00	Closed fracture axis, transverse process
Vertebrae	Syu1500	[X]Fracture of other specified cervical vertebra
Vertebrae	S101A00	Open fracture axis, odontoid process
Vertebrae	S100E00	Closed fracture axis, posterior arch
Vertebrae	S100M00	Closed fracture cervical vertebra, posterior arch
Vertebrae	S104600	Closed fracture lumbar vertebra, tricolumnar
Vertebrae	S100J00	Closed fracture cervical vertebra, spondylolysis
Vertebrae	S104500	Closed fracture lumbar vertebra, posterior arch
Vertebrae	S102200	Closed fracture thoracic vertebra, spondylolysis
Vertebrae	S101211	C2 vertebra open fracture without spinal cord lesion
Vertebrae	S107100	Open vertical fracture of sacrum
